# An Indigenous Food Is Medicine Intervention: The MUTTON-HF Randomized Clinical Trial

**DOI:** 10.1001/jamainternmed.2026.2879

**Published:** 2026-07-27

**Authors:** Lauren A. Eberly, Carmen George, Sharon Sandman, Denee Bex, Karianne Jones, Asia Yazzie, Leah Gray, Larissa Morgan, Ada Tennison, Conor Williams, Matt Chandra, Rebecca Wickre, Bennett Wickre, Mackenzie Bolas, Remi Welbel, Delaney Ignace, Benjamin Feliciano, Stacy Hammer, DezBaa Damon-Mallette, Erica Lindsey, Paula Mora, Aijun Ye, Maricruz Merino, Enrique F. Schisterman, Sonya S. Shin

**Affiliations:** 1 Gallup Indian Medical Center, Indian Health Service, Gallup, New Mexico; 2 Division of Cardiovascular Medicine, Department of Medicine, Hospital of the University of Pennsylvania, Philadelphia; 3 Penn Cardiovascular Outcomes, Quality, and Evaluative Research Center, Cardiovascular Institute, University of Pennsylvania, Philadelphia; 4 Penn Cardiovascular Center for Health Equity and Social Justice, University of Pennsylvania, Philadelphia; 5 Leonard Davis Institute of Health Economics, University of Pennsylvania, Philadelphia; 6 Division of Global Health Equity, Brigham and Women’s Hospital, Boston, Massachusetts; 7 Tumbleweed Nutrition, LLC, Farmington, New Mexico; 8 Tocabe Inc, Denver, Colorado; 9 Harvard School of Medicine, Boston, Massachusetts; 10 Harvard College, Boston, Massachusetts; 11 Office of Quality, Division of Innovations and Improvement, Indian Health Service Headquarters, Rockville, Maryland; 12 Office of Clinical and Preventive Services, Indian Health Service Headquarters, Rockville, Maryland; 13 Department of Biostatistics, Epidemiology and Informatics, University of Pennsylvania, Philadelphia; 14 Department of Global Health and Social Medicine, Harvard Medical School, Boston, Massachusetts

## Abstract

**Importance:**

Nutrition insecurity is a major driver of poor cardiovascular health in Indigenous communities. Medically tailored meals that reclaim traditional foods may improve heart failure outcomes and quality of life. Community-based participatory methods were used to design Medically Utilized Tailored Traditional Foods to Optimize Nutrition in Heart Failure (MUTTON-HF), a culturally and medically tailored meal program incorporating traditional Navajo foods and recipes.

**Objective:**

To determine the efficacy of a culturally and medically tailored meal program on the incidence of hospitalizations and emergency department visits.

**Design, Setting, and Participants:**

This pragmatic, open-label randomized clinical trial was conducted from May to November 2025 at 2 Indian Health Service sites in rural Navajo Nation. Eligible patients were adults with heart failure who were receiving care at the study sites and had a hospitalization or emergency department visit during the last 12 months. All patients were followed for 12 weeks for outcomes, death, and adverse events. The data were analyzed from December 2025 to February 2026.

**Intervention:**

Patients were randomized in a 1:1 ratio to 8 weeks of a culturally and medically tailored meal program or usual dietary advice.

**Main Outcomes and Measures:**

The primary end point was the proportion of patients with an all-cause hospitalization or emergency department visit within 90 days. Secondary outcomes included hospitalizations or emergency department visits separately, and for heart failure specifically, and change in Kansas City Cardiomyopathy Questionnaire scores, food insecurity, financial strain, blood pressure, and weight from enrollment to 8 weeks.

**Results:**

A total of 206 patients (mean [SD] age, 65.8 [14.2] years; 87 female individuals [42%] and 119 male individuals [58%]; 203 American Indian individuals [99%], 2 American Indian or Alaskan Native and White individuals [0.97%], and 1 White individual [0.03%]; ejection fraction, 48%) were randomized. The primary outcome was significantly less frequent in the intervention arm (43 [40.6%] vs 57 [57.0%]; relative risk, 0.72; 95% CI, 0.54-0.96; *P* = .02), which was driven mainly by reduced hospitalizations (13 [12.3%] vs 26 [26.0%]). There was a lower incidence of heart failure hospitalizations specifically (4 [3.8%] vs 13 [13.0%]). The Kansas City Cardiomyopathy Questionnaire score and food security measures had significantly greater increases in the intervention arm while measures of financial strain, weight, and blood pressure significantly decreased in the intervention arm. Adverse events were uncommon.

**Conclusions and Relevance:**

In this randomized clinical trial, an Indigenous Food is Medicine intervention reduced the incidence of hospitalization and emergency department visits among patients with heart failure. Community-based interventions that leverage protective assets of Native communities are needed to advance Indigenous health.

**Trial Registration:**

ClinicalTrials.gov Identifier: NCT06549699

## Key Points

**Question:**

Does an Indigenous culturally and medically tailored meal program reduce the incidence of hospitalizations and emergency department visits among patients with heart failure?

**Findings:**

In this randomized clinical trial that included 206 adults in rural Navajo Nation, the proportion of patients with a hospitalization or emergency department visit was significantly lower among those in the intervention arm compared with usual care (40.6% vs 57.0%).

**Meaning:**

This trial found that culturally and medically tailored meal programs can improve clinical outcomes for patients with heart failure in rural and tribal settings and advance Indigenous cardiovascular health.

## Teaser

This randomized clinical trial examines the efficacy of a culturally and medically tailored meal program on the incidence of hospitalizations and emergency department visits among Indigenous communities.

## Introduction

Cardiovascular health inequities among Indigenous communities are rooted in the historic and enduring effects of settler colonialism.^1,2,3,4^ Nutrition insecurity, which is endemic in many Native communities, is a major driver of disproportionate rates of cardiovascular disease and poor cardiovascular health.^4,5,6^ Genocide, broken treaty obligations, land theft, forced migration, environmental injustice, and exclusionary governmental policies have fueled nutrition insecurity in Indigenous communities.^1,2,3,4^

Advancing Indigenous cardiovascular health requires holistic solutions that leverage the protective assets of Native communities.^1^ Many Indigenous epistemologies include the concept of Food is Medicine.^1,7,8,9,10,11^ In Diné (Navajo) culture, traditional foods are considered sacred and central to healing. Food is Medicine interventions can improve health and well-being by addressing the interconnected determinants of Indigenous health through reclaiming traditional precontact foods, strengthening cultural connections, fostering intergenerational transfer of knowledge, and reinforcing connections to ancestral and other lands.^12,13,14,15,16,17,18,19,20,21,22,23^ In Western science, diet plays an important role in heart failure (HF) outcomes, and there is increasing evidence that medically tailored meals (MTMs) may improve cardiovascular outcomes and quality of life.^24,25,26,27^

Given this, we used community-based participatory methods to design Medically Tailored Traditional Food to Optimize Nutrition in Heart Failure (MUTTON-HF), a locally and Native-sourced MTM delivery program incorporating traditional Navajo foods and recipes.^28^ A pilot feasibility, single-arm, nonrandomized study of a 4-week intervention among patients with HF in Navajo Nation demonstrated feasibility and acceptability.^23^ Therefore, we conducted a randomized clinical trial of MUTTON-HF to test the hypothesis that a locally sourced Indigenous culturally and medically tailored meal (CMTM) program would lower rates of hospitalizations and emergency department (ED) visits compared with usual care.

## Methods

### Study Design

The design and methods of MUTTON-HF have previously been described.^29^ Additionally, the full protocol is available through the study website (NCT06549699) and Supplement 1.^28,30^ The data were analyzed from December 2025 to February 2026.

MUTTON-HF was a 12-week, pragmatic, open-label randomized clinical trial that was conducted at 2 Indian Health Service (IHS) sites. This study was part of an IHS Office of Quality Innovations Award and was funded by the American Heart Association Healthcare by Food initiative. This trial was conducted according to the principles of the Declaration of Helsinki and approved by the Navajo Nation Human Research Review Board (NNR 24.525) and Mass General Brigham. Patient consent was obtained either in person or verbally by phone. We followed the Consolidated Standards of Reporting Trials (CONSORT) guidelines. Data are owned by Navajo Nation and can be requested from the Navajo Nation Human Research Review Board.

### Study Setting

This study was conducted in rural Navajo Nation at 2 IHS ambulatory clinics (the largest IHS facility serving Navajo Nation and a smaller satellite clinic on the reservation^31,32^) with a combined catchment area of a larger than 75-mile radius.^32^ Of the 27 000 square miles of land in the Navajo Nation, the largest US reservation, there are 13 grocery stores and approximately 110 smaller stores that are limited in fresh produce and healthy food options.^33^

### Inclusion and Exclusion Criteria

We included patients with a diagnosis of HF who were receiving care at either study facility (defined as at least 1 clinical encounter and 1 prescription during the last 12 months) and had an ED visit and/or hospitalization during the last 12 months. Patients who were receiving hospice care, living at a skilled nursing or short-term rehabilitation facility, or were unable to provide consent were excluded.

### MUTTON-HF Intervention

The MUTTON-HF intervention, which was designed with community-based participatory methods, has been previously described.^23,28,29^ Meals were created by a local Navajo dietitian and culinary expert (D.B.) to incorporate traditional Navajo foods and recipes (eFigure 1 in Supplement 2)^34^ and ensure they met American Heart Association’s sodium-restricted Dietary Approaches to Stop Hypertension guidelines and American Heart Association Heart-Check Food Certification Program nutrition requirements.^35,36,37^ We partnered with local Navajo farmers and ranchers to source meat and produce and with Tocabe, a Native-run kitchen, to produce the medically tailored frozen meals, practicing whole animal utilization and Native first supply chain when possible.^19,38^ We purchased meals for $12 per meal, which did not include meal research and development, delivery, and distribution costs. Because most patients lacked physical addresses for mail delivery, a distribution system was established with 2 primary pick-up sites (a community pantry and the satellite clinic) and delivery to miniature food hubs for local pick-up or directly to home,^23^ with support from Public Health Nurses and tribal Community Health Representatives. Patients received any needed household infrastructure to participate in the intervention, including microwaves, freezers, and (if lacking electricity) a propane-fueled refrigerator, stove, and propane supply.

Patients in the intervention arm received 14 CMTMs weekly (2 meals daily) for 60 days. A sample 1-week menu of meals with additional meal details is shown in eTable 1 in Supplement 2 and nutrient fact panels for a selection of meals in eTable 2 in Supplement 2. The intervention and control arms received usual care, a booklet with nutritional recommendations, and a cooking class at the end of the outcome assessment period. Given community preferences and ethical concerns, patients in the control arm were offered 60 days of meals after the assessment period. As a pragmatic trial, study participants could decline survey and laboratory work at any point but continue study enrollment and receipt of meals.

### Primary and Secondary Outcomes

The primary outcome was the proportion of patients with a hospitalization or ED visit for any cause within 90 days. Secondary outcomes included all-cause hospitalizations, all-cause ED visits, HF hospitalizations, and ED visits for HF, as well as changes in the following measures at 60 days from baseline: blood pressure, weight, body mass index (calculated as weight in kilograms divided by height in meters squared), Kansas City Cardiomyopathy Score (KCCS, including summary, physical limitation, symptom frequency, quality of life, and social limitation scores)^39,40^; albumin, prealbumin, creatinine, N-terminal pro–B-type natriuretic peptide, hemoglobin A_1c_, cholesterol (total, low-density lipoprotein and high-density lipoprotein cholesterol, and triglycerides), and C-reactive protein levels; and urine albumin: creatinine ratios. Patients used a home blood pressure cuff (Omron 5 series) and scale for blood pressure and weight measurements.

We also assessed diet quality using the 10-item Dietary Screener Questionnaire,^41,42^ with an added question to assess traditional Diné food intake at 60 days, as well as changes at 60 days in food insecurity using the US Department of Agriculture Adult Food Security: Six Item Short Form Module,^43,44^ Indigenous Nourishment Scale (Measure A),^45^ and financial stress using the Financial Stress Scale.^46^ We also measured baseline cultural connectedness using the Cultural Connectedness Scale, short version.^47,48,49^

### Intervention Indicators and Exploratory Outcomes

We evaluated the proportion of meals that included Native-sourced and Diné-sourced ingredients specifically, as well as the percentage of meals successfully distributed to the patient. Patient satisfaction was evaluated by patient rating of the program from 1 to 10, which was used to calculate the Net Promoter Score postintervention. We assessed adherence based on self-reported meal consumption and delivery/pick up logs. Adverse events were assessed through electronic health record (EHR) review. Additional exploratory outcomes included weekly physical activity, as well as patient-reported health status at 60 days.

### Randomization and Blinding

Participants were randomized in a 1:1 ratio to the intervention or usual care using permuted block randomization (block size = 6), stratified by left ventricular ejection fraction (<50% vs ≥50%), age (<65 vs ≥65 years), and sex. For participants from the same household, the first enrollee was randomized and subsequent members were assigned to the same arm to avoid contamination. Neither participants nor clinicians were masked to the allocation. Outcomes were ascertained from patient surveys and the EHR. Data analysts and study staff who were extracting outcomes from the EHR were masked to treatment assignment.

### Statistical Analysis

The primary analysis followed the intention-to-treat principle, with all randomized participants analyzed in the groups to which they were assigned, regardless of adherence or protocol deviations. Relative risk estimates were generated with log-binomial generalized estimating equation models to account for clustering for those in the same household (n = 3). Between-group differences in the primary outcome and dichotomous secondary outcomes were assessed using χ^2^ tests or the Fischer exact test, as well as 2-sample *t* tests for continuous variables. Within-group randomization to week 8 changes in continuous outcomes, including laboratory evaluation and biomarkers, Indigenous nourishment scale scores, financial stress scale scores, and KCCQ scores, were evaluated using the Wilcoxon signed-rank test.

### Sample Size Calculation

Based on a baseline event rate of comparable patients at the study sites and prior data on the effectiveness of MTM interventions in other settings, we hypothesized a reduction from a baseline of 29% to 13% in the incidence of hospitalizations or ED visits within 90 days,^24^ resulting in a sample size of 204 patients to detect a significant difference, with 80% power and an α error rate of .05, assuming no loss to follow-up given the short study duration and high retention rates in our previous trials.^50^

### Prespecified Subgroup Analyses

We performed prespecified subgroup analyses of the primary outcome based on left ventricular ejection fraction (LVEF, <50% vs ≥50%), sex (male compared with female), age (<65 vs ≥65 years), and food insecurity (food insecure vs food secure). We also evaluated the change in weight among those with baseline obesity (body mass index ≥30). Given the low risk of the intervention, prior pilot data supporting the safety of the intervention,^23^ and short duration of the intervention, no interim safety analyses were performed.

## Results

**Figure 1.**
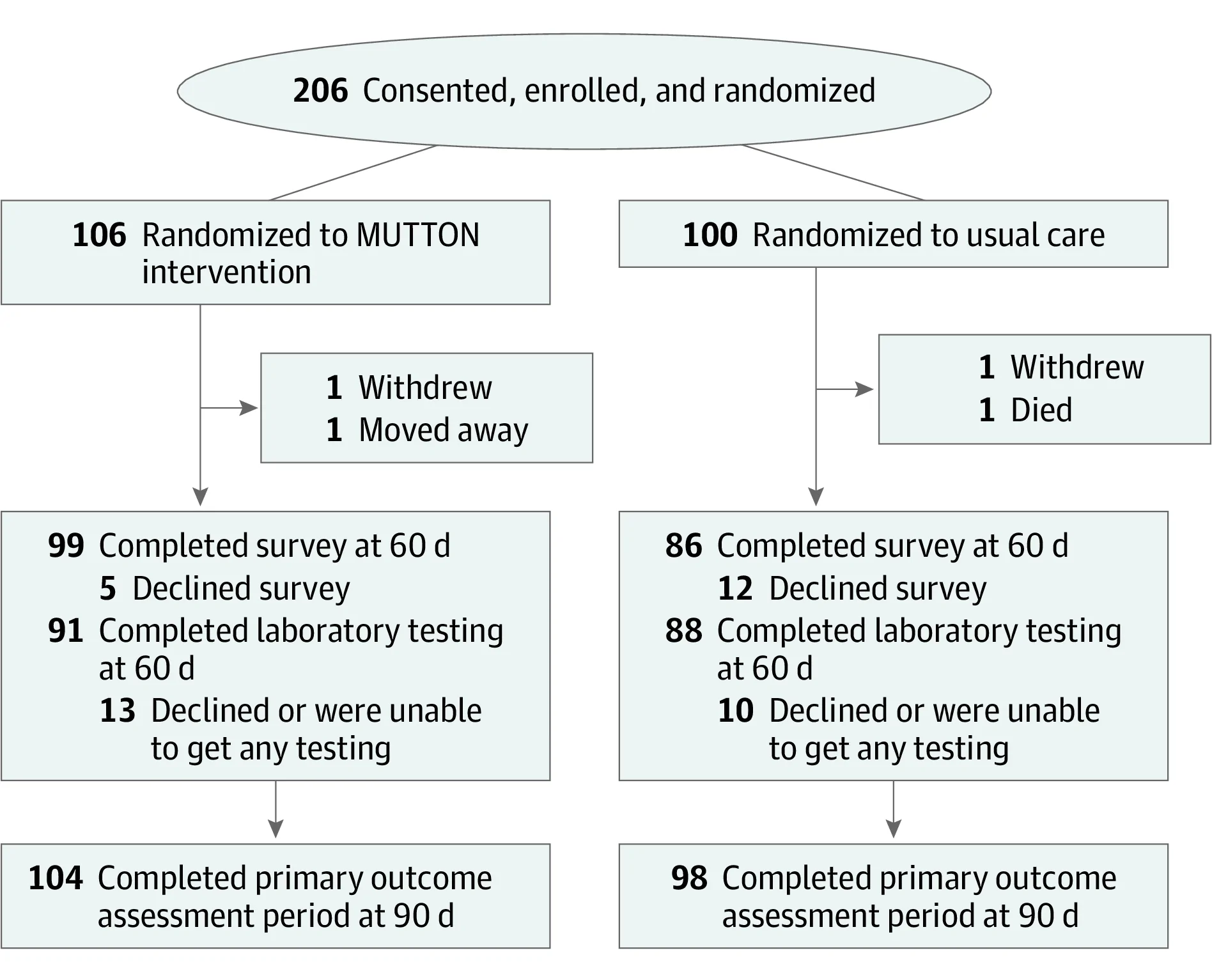
Study Design and Consort Diagram of the Medically Utilized Tailored Traditional Foods to Optimize Nutrition in Heart Failure (MUTTON-HF) Trial Due to study design and community preference, patients could opt out of laboratory evaluation or surveys at any point but continue in the study. HF indicates heart failure.

A total of 206 patients were enrolled (Figure 1). Three participants were not randomized but were assigned to the intervention arm based on assignment of a previously enrolled household member.

At 90 days, 1 participant had died, 2 had withdrawn consent, and 1 had moved out of the area. Therefore, 202 patients were followed up for 90 days for the primary outcome assessment. The proportion of participants who declined surveys and all laboratory evaluations were 28 (13.6%) for the baseline survey, 17 (8.3%) for the 60-day survey, 18 (8.7%) for the baseline laboratory evaluation, and 23 (11.2%) for the 60-day laboratory evaluation.

### Baseline Characteristics

As shown in Table 1,^51^ the cohort was predominantly American Indian (203 [98.5%]), male (119 [57.8%]; 87 female individuals [42.2%]), with a mean (SD) age of 65.8 (14.2) years and mean (SD) LVEF of 48.1% (13.8%). The residential catchment area spanned 120 miles north to south and 155 miles from east to west across 2 states (eFigure 2 in Supplement 2). There were high rates of baseline obesity (94 [45.6%]), type 2 diabetes (135 [65.5%]) or prediabetes (43 [20.9%]), hypertension (148 [71.8%]), and dyslipidemia (133 [64.6%]). Additional baseline characteristics and baseline medications by group assignment are shown in eTable 3 in Supplement 2, as well as for those with complete vs missing survey or biomarker data in eTables 4 and 5 in Supplement 2. The baseline mean (SD) KCCQ summary score was 58.4 (23.8). A total of 110 participants (53.4%) had food insecurity, with a mean (SD) consumption of fruits of 0.9 (0.5) cups per day and vegetables (excluding fried potatoes) of 1.6 (0.6) cups per day.

**Table 1.** Baseline Characteristics of 206 Participants in the MUTTON-HF Study Cohort

Characteristic	No. (%)	
	Intervention (early meals group) (n = 106)	Control arm (n = 100)
Demographic		
Age, mean (SD), y	65 (14.1)	66.7 (14.2)
Sex		
Female	45 (42.5)	42 (42.0)
Male	61 (57.5)	58 (58.0)
Race		
American Indian or Alaskan Native	104 (98.1)	99 (99.0)
American Indian or Alaskan Native and White	2 (1.9)	0
White	0	1 (1.0)
Tribal affiliation		
Navajo	101 (95.3)	94 (94.9)
Zuni	1 (0.9)	1 (1.0)
Navajo and Zuni	1 (0.9)	0 (0)
Other	3 (2.8)	4 (4.0)
None	0	1
Hispanic ethnicity	1 (0.9)	2 (2.0)
Cultural Connectedness score total, mean (SD)	27.1 (7.6)	24.9 (8.1)
Indigenous Nourishment scale score, mean (SD)	3.8 (1.5)	3.8 (1.4)
Diet quality and characteristics		
Responsible for some or most of the cooking	66 (62.2)	49 (49.0)
Daily servings of fruits and vegetables (based on DSQ-10), mean (SD), cups	2.4 (0.9)	2.4 (1.0)
Socioeconomic and household characteristics		
USDA food security status		
High food security	36 (37.9)	30 (37.0)
Low food security	39 (41.1)	35 (43.2)
Very Low security	20 (21.1)	16 (19.8)
Financial Stress score, mean (SD)	7.1 (2.7)	7.3 (2.8)
Working microwave	85 (80.2)	69 (69.0)
Working stove	88 (83.0)	78 (78.0)
Working freezer	84 (79.2)	72 (72.0)
Working refrigerator	87 (82.1)	76 (76.0)
Reliable electricity	86 (81.1)	77 (77.0)
Running tap water	78 (73.6)	70 (70.0)
Water insecure^a^	44 (41.9)	51 (50.5)
Enrollment in food assistance programs^b^		
Supplemental Nutrition Assistance Program/food stamps	38 (35.8)	37 (37.0)
Commodities	4 (3.8)	5 (5.0)
WIC	4 (3.8)	3 (3.0)
Other	44 (41.5)	31 (31.0)
Clinical characteristics		
Weight, mean (SD), lb	192.4 (65.0)	190.1 (58.2)
BMI, mean (SD)	31 (8.6)	31 (8.7)
Systolic blood pressure, mean (SD), mm Hg	132.8 (20.7)	128.2 (18.0)
Diastolic blood pressure, mean (SD), mm Hg	78.1 (14.6)	74.5 (12.6)
Type of heart failure		
Ischemic	27 (25.5)	28 (28.0)
Nonischemic	47 (44.3)	30 (30.0)
Mixed	3 (2.8)	3 (3.0)
Unknown	29 (27.4)	39 (39.0)
Left ventricular ejection fraction, mean (SD), %	48.1 (13.8)	48.1 (14.0)
Comorbidities		
Hypertension	68 (64.2)	80 (80.0)
Atrial fibrillation	21 (19.8)	24 (24.0)
Coronary artery disease	45 (42.5)	47 (47.0)
CKD	33 (31.1)	39 (39.0)
CKD and receiving hemodialysis	4 (3.8)	6 (6.0)
Type 2 diabetes	68 (64.2)	67 (67)
Hyperlipidemia	67 (63.2)	66 (66)
Obesity	45 (42.5)	49 (49)
Prediabetes	21 (19.8)	22 (22)
Obstructive sleep apnea	25 (23.6)	24 (24)
KCCQ summary score total, mean (SD)	59.9 (24.5)	56.5 (22.9)
KCCQ physical limitation score	56.1 (29.1)	54.7 (27.9)
KCCQ symptom frequency score	68.5 (28.5)	62.4 (28.2)
KCCQ quality of life score	56.2 (30.7)	52.6 (28.3)
KCCQ social limitation score	58 (31.4)	55.9 (31.7)

Abbreviations: BMI, body mass index (calculated as weight in kilograms divided by height in meters squared); CKD, chronic kidney disease; DSQ, Dietary Screener Questionnaire; KCCQ-Kansas City Cardiomyopathy Questionnaire; MUTTON-HF, Medically Utilized Tailored Traditional Foods to Optimize Nutrition in Heart Failure; USDA, US Department of Agriculture; WIC, Special Supplemental Nutrition Program for Women, Infants, and Children.

^a^ Water insecurity was determined by validated water security indicators with having any of the following responses being consistent with water insecurity: answering “no” to “Do you have running (tap) water”; answering “disagree” to “My tap water at home is safe to drink” or “My tap water at home is safe to cook with”; or “I never drink tap water” when asked “When you drink tap water, what is the main source of the tap water?”^51^

^b^ During prior 12 months.

For those in the intervention group, a mean (SD) of 6.0 (2.7) of 8.0 weekly meal boxes were received (mean [SD] proportion of meal boxes received by patients, 75.6% [33.2%]) (eTable 6 in Supplement 2). Seventy-six participants (75.0%) reported that they ate most or all meals. Participants rated the program a mean (SD) of 8.3 (2.3) of 10, with a Net Promoter score of 39%. Household appliances provided by group are shown in eTable 7 in Supplement 2. In the intervention arm, 17 (16.0%) received at least 1 household appliance, and 6 (5.7%) did not have stable electricity and received a propane-powered fridge. All meals (100%) were sourced with Native produce and meat, with 47.3% with local Navajo farmer or rancher ingredients specifically.

### Primary and Secondary Outcomes

The primary outcome occurred significantly less frequently in the intervention arm vs the control arm (40.6% vs 57.0%; relative risk [RR], 0.72; 95% CI, 0.54-0.96; *P* = .02) (Table 2). Differences in the primary outcome were driven mainly by reduced hospitalizations (12.3% vs 26.0%; RR, 0.48; 95% CI, 0.26-0.89) (Table 2). Hospitalizations for HF were lower in the intervention arm (3.8% vs 13.0%; RR, 0.29; 95% CI, 0.10-0.87).

**Table 2.** Primary and Secondary Clinical Outcome and Events

Outcome	No. (%)		Relative risk (95% CI)
	Intervention arm (n = 106)	Control group (n = 100)	
Primary outcome			
Hospitalization or ED visit (all-cause)	43 (40.6)	57 (57.0)	0.72 (0.54-0.96)
Secondary outcomes			
Hospitalization	13 (12.3)	26 (26.0)	0.48 (0.26-0.89)
ED visit	34 (32.1)	44 (44.0)	0.73 (0.51-1.05)
HF hospitalization	4 (3.8)	13 (13.0)	0.29 (0.10-0.87)
ED visit for HF	1 (0.9)	1 (1.0)	0.95 (0.06-15.05)
Hospitalization or ED visit for HF	4 (3.8)	13 (13.0)	0.29 (0.10-0.87)

Abbreviations: ED, emergency department; HF, heart failure.

Within-group and between group differences in secondary outcomes and clinical biomarkers are summarized in Table 3. There were significant increases in the daily cups of fruits and vegetables based on the Dietary Screener Questionnaire–10 in the intervention group, which differed compared with changes in the control group (between-group difference of 0.24 cups daily).

**Table 3.** Within- and Between-Group Differences in Patient-Reported Outcomes and Clinical Biomarkers

Outcome	Arm	Mean (SD)		Change (95% CI)^a^	Change, % (95% CI)^a^
		Baseline	Postbaseline		
Weight	Food arm	187.57 (52.71)	184.05 (48.55)	−3.52 (−6.93 to −1.33)	−1.88 (−3.65 to −0.73)
	Control	190.12 (58.25)	192.89 (58.63)	2.77 (−0.95 to 6.06)	1.46 (−0.49 to 3.26)
	Food vs control			−6.29 (−10.83 to −2.05)	−3.33 (−5.75 to −1.10)
Systolic blood pressure	Food arm	132.78 (20.71)	127.55 (19.55)	−5.23 (−8.77 to −1.88)	−3.94 (−6.58 to −1.44)
	Control	128.23 (18.03)	129.71 (19.83)	1.48 (−3.40 to 6.23)	1.16 (−2.62 to 4.96)
	Food vs control			−6.72 (−12.58 to −0.85)	−5.10 (−9.61 to −0.59)
Diastolic blood pressure	Food arm	78.11 (14.64)	75.6 (11.23)	−2.52 (−4.95 to −0.03)	−3.22 (−6.20 to −0.03)
	Control	74.54 (12.63)	75.22 (11.52)	0.68 (−1.89 to 2.88)	0.92 (−2.47 to 3.94)
	Food vs control			−3.20 (−6.60 to 0.31)	−4.14 (−8.54 to 0.43)
Indigenous Nourishment scale	Food arm	3.81 (1.47)	4.53 (1.06)	0.72 (0.47 to 1.08)	18.98 (11.56 to 30.47)
	Control	3.76 (1.41)	4.19 (1.08)	0.43 (0.13 to 0.79)	11.33 (2.94 to 22.19)
	Food vs control			0.30 (−0.13 to 0.71)	7.65 (−4.98 to 20.19)
Financial Strain scale	Food arm	7.13 (2.66)	6.47 (2.64)	−0.66 (−1.21 to −0.16)	−9.26 (−16.26 to −2.30)
	Control	7.25 (2.82)	7.07 (2.77)	−0.18 (−0.82 to 0.40)	−2.48 (−10.86 to 5.77)
	Food vs control			−0.47 (−1.22 to 0.38)	−6.77 (−16.87 to 4.77)
KCCQ summary score	Food arm	59.94 (24.55)	61.67 (24.73)	1.74 (−2.84 to 5.90)	2.90 (−4.56 to 10.36)
	Control	56.51 (22.93)	53.15 (22.02)	−3.35 (−7.76 to 1.06)	−5.93 (−13.19 to 2.01)
	Food vs control			5.09 (−1.13 to 11.35)	8.83 (−1.98 to 19.53)
KCCQ-12 physical limitation	Food arm	56.05 (29.09)	57.95 (29.85)	1.89 (−3.98 to 8.20)	3.38 (−6.78 to 15.93)
	Control	54.71 (27.89)	48.78 (30.03)	−5.93 (−12.61 to 0.94)	−10.85 (−21.68 to 1.92)
	Food vs control			7.83 (−1.28 to 16.97)	14.23 (−2.86 to 30.86)
KCCQ-12 symptom frequency	Food arm	68.54 (28.54)	71.25 (26.72)	2.71 (−1.52 to 7.57)	3.96 (−2.27 to 11.77)
	Control	62.38 (28.21)	64.87 (26.28)	2.49 (−3.96 to 9.09)	3.99 (−5.69 to 15.58)
	Food vs control			0.22 (−7.52 to 8.69)	−0.03 (−12.84 to 13.50)
KCCQ-12 quality of life	Food arm	56.19 (30.68)	56.44 (28.59)	0.25 (−5.25 to 5.47)	0.45 (−8.63 to 10.08)
	Control	52.62 (28.28)	50.71 (26.35)	−1.91 (−8.20 to 5.01)	−3.64 (−14.55 to 10.95)
	Food vs control			2.16 (−6.68 to 10.39)	4.08 (−12.78 to 19.21)
KCCQ-12 social limitation	Food arm	57.98 (31.39)	61.71 (32.52)	3.73 (−3.16 to 10.17)	6.44 (−4.82 to 18.57)
	Control	55.92 (31.71)	45.47 (31.38)	−10.45 (−18.20 to −2.37)	−18.68 (−30.51 to −4.41)
	Food vs control			14.18 (3.65 to 24.72)	25.12 (7.41 to 42.74)
Food Insecurity score	Food arm	2.36 (2.01)	1.72 (1.74)	−0.64 (−1.02 to −0.28)	−27.17 (−40.04 to −13.19)
	Control	2.44 (2.03)	2.29 (1.96)	−0.16 (−0.57 to 0.25)	−6.49 (−22.02 to 10.27)
	Food vs control			−0.48 (−1.08 to 0.05)	−20.68 (−43.55 to −0.29)
**DSQ-10**					
Daily cups of fruits and vegetables (including fried potatoes)	Food arm	2.5 (0.85)	2.63 (0.86)	0.13 (−0.02 to 0.29)	5.05 (−0.90 to 11.59)
	Control	2.53 (1)	2.42 (0.82)	−0.11 (−0.29 to 0.06)	−4.16 (−11.05 to 2.28)
	Food vs control			0.23 (0.00 to 0.47)	9.21 (0.17 to 18.42)
Daily cups of fruits and vegetables (not including fried potatoes)	Food arm	2.4 (0.88)	2.52 (0.88)	0.13 (−0.03 to 0.30)	5.24 (−1.41 to 12.92)
	Control	2.42 (1.01)	2.31 (0.82)	−0.12 (−0.31 to 0.05)	−4.76 (−12.32 to 2.09)
	Food vs control			0.24 (0.01 to 0.49)	10.00 (0.35 to 20.08)
**Clinical biomarkers**					
Total cholesterol, mg/dL	Food arm	139.64 (41.68)	139.49 (43.67)	−0.15 (−6.27 to 6.24)	−0.11 (−4.46 to 4.48)
	Control	127.33 (41.28)	133.68 (39.18)	6.35 (−1.18 to 14.28)	4.98 (−0.91 to 11.46)
	Food vs control			−6.50 (−16.77 to 3.54)	−5.09 (−13.06 to 2.44)
HDL, mg/dL	Food arm	47.58 (16.24)	45.87 (13.63)	−1.71 (−3.89 to 0.26)	−3.60 (−7.81 to 0.55)
	Control	45.43 (12.76)	45.44 (11.14)	0.01 (−2.15 to 2.01)	0.02 (−4.66 to 4.54)
	Food vs control			−1.72 (−4.54 to 1.39)	−3.62 (−9.59 to 2.87)
LDL, mg/dL	Food arm	74.24 (34.84)	73.21 (35.77)	−1.02 (−5.80 to 3.54)	−1.38 (−7.72 to 4.85)
	Control	63.88 (33.02)	70.08 (31.02)	6.19 (0.27 to 13.29)	9.69 (0.32 to 22.96)
	Food vs control			−7.21 (−15.18 to 0.38)	−11.07 (−25.40 to 0.15)
Triglycerides, mg/dL	Food arm	155.13 (113.09)	155.7 (118.52)	0.57 (−15.65 to 20.88)	0.37 (−9.38 to 14.62)
	Control	144.08 (140.88)	140.74 (92.52)	−3.35 (−35.55 to 14.48)	−2.32 (−19.38 to 11.62)
	Food vs control			3.91 (−20.04 to 46.41)	2.69 (−13.50 to 28.32)
Creatinine, mg/dL	Food arm	1.41 (1.29)	1.54 (1.87)	0.13 (−0.03 to 0.63)	9.39 (−2.31 to 39.01)
	Control	1.42 (1.14)	1.70 (1.70)	0.28 (0.13 to 0.74)	20.08 (9.21 to 52.78)
	Food vs control			−0.15 (−0.52 to 0.18)	−10.69 (−36.41 to 12.07)
Prealbumin, mg/dL	Food arm	19.51 (4.76)	19.35 (5.55)	−0.16 (−1.16 to 0.91)	−0.81 (−5.92 to 4.87)
	Control	17.93 (6.07)	18.17 (5.61)	0.24 (−1.13 to 1.62)	1.36 (−6.02 to 9.40)
	Food vs control			−0.40 (−2.02 to 1.36)	−2.17 (−11.02 to 7.25)
Albumin, g/dL	Food arm	3.95 (0.46)	3.96 (0.50)	0.02 (−0.11 to 0.09)	0.43 (−2.62 to 2.37)
	Control	3.78 (0.48)	3.91 (0.45)	0.13 (0.05 to 0.20)	3.46 (1.36 to 5.50)
	Food vs control			−0.11 (−0.25 to 0.00)	−3.03 (−6.54 to −0.01)
Urine albumin:creatinine ratio, mg/g	Food arm	614.08 (1373.83)	667.43 (2329.01)	53.35 (−296.81 to 1149.30)	8.69 (−42.82 to 319.24)
	Control	632.13 (1354.63)	580.38 (1656.6)	−51.75 (−328.20 to 541.00)	−8.19 (−46.37 to 138.54)
	Food vs control			105.10 (−452.30 to 895.93)	16.87 (−72.21 to 227.93)
CRP, mg/dL	Food arm	0.64 (1.25)	0.83 (1.59)	−0.04 (−1.59 to 0.61)	−4.44 (−67.07 to 137.72)
	Control	0.99 (1.99)	0.9 (1.50)	−0.71 (−2.52 to 0.22)	−41.46 (−73.17 to 41.07)
	Food vs control			0.67 (−0.88 to 2.36)	37.01 (−101.90 to 202.53)
HbA_1c_, %	Food arm	7.15 (2.23)	7.07 (1.90)	−0.07 (−0.39 to 0.18)	−1.05 (−5.22 to 2.55)
	Control	7.20 (1.84)	6.9 (1.54)	−0.29 (−0.67 to 0.03)	−4.08 (−8.97 to 0.57)
	Food vs control			0.22 (−0.21 to 0.66)	3.03 (−2.99 to 8.98)
NT-pro BNP, pg/mL	Food arm	3476.67 (11394.35)	3727.7 (12333.44)	251.03 (−396.81 to 1229.13)	7.22 (−15.83 to 35.81)
	Control	2707.65 (5691.98)	4462.55 (12788.64)	1754.90 (173.06 to 5282.35)	64.81 (1.82 to 223.97)
	Food vs control			−1503.87 (−5081.87 to 281.88)	−57.59 (−221.19 to 10.46)
Potassium, mEq/L	Food arm	4.12 (0.50)	4.14 (0.55)	0.02 (−0.08 to 0.16)	0.59 (−1.93 to 3.92)
	Control	4.07 (0.45)	4.06 (0.37)	−0.01 (−0.10 to 0.08)	−0.21 (−2.38 to 1.99)
	Food vs control			0.03 (−0.10 to 0.18)	0.80 (−2.49 to 4.53)
Sodium, mEq/L	Food arm	138.42 (3.44)	138.03 (3.64)	−0.39 (−1.07 to 0.43)	−0.28 (−0.77 to 0.31)
	Control	138.59 (3.52)	138.41 (3.8)	−0.18 (−0.85 to 0.54)	−0.13 (−0.61 to 0.39)
	Food vs control			−0.21 (−1.24 to 0.80)	−0.15 (−0.89 to 0.57)

Abbreviations: CRP, C reactive protein; DSQ, Dietary Screener Questionnaire; HbA_1c_, hemoglobin A_1c_; HDL, high-density lipoprotein; KCCQ, Kansas City Cardiomyopathy Questionnaire (12-item); LDL, low-density lipoprotein; NT-pro BNP, N-terminal pro-B-type natriuretic peptide.

SI conversion factors: to convert albumin to g/L, multiply by 10; for cholesterol (LDL and HDL) to mmol/L, multiply by 0.0259; for creatinine to µmol/L, multiply by 88.4; for HbA_1c_ to the proportion of total hemoglobin, multiply by 0.01; for potassium to mmol/L, multiply by 1; for prealbumin to mg/L, multiply by 10; for sodium to mmol/L, multiply by 1; for triglycerides to mmol/L, multiply by 0.0113.

^a^ 95% CIs determined using bootstrap methods.

Food insecurity significantly decreased in the intervention arm from baseline to 60 days, and changes differed from the control group (−0.48 between-group difference). The Indigenous Nourishment Scale score increased in both groups from baseline to 60 days.

The KCCQ total score, as well as the physical limitation and social limitation subcomponents, improved in the intervention group from baseline to 60 days, which differed from changes in the control group (between-group difference in KCCQ summary score of 5.09, change of 8.83%).

Weight decreased in the intervention group and increased in the control group, with a between-group difference of −6.3 pounds (3.33% change). Among those with baseline obesity, the between-group difference was −7.31 lbs. Systolic and diastolic blood pressure decreased from baseline in the intervention arm, with increases in the control arm (between-group difference of −6.7 mm Hg systolic and −3.2 mm Hg diastolic). Decreases in the financial strain score from baseline to 60 days were higher in the intervention group compared with the control arm (−0.47 [−6.8% change] in the intervention vs control arm).

N-terminal pro–B-type natriuretic peptide levels increased less in the intervention group compared with the control group from baseline to 60 days. Creatinine levels decreased in the intervention group compared with the control group. There were no other differences in biomarkers between groups. There were significant differences in adverse events between arms (eTable 8 in Supplement 2).

### Exploratory and Subgroup Analyses

**Figure 2.**
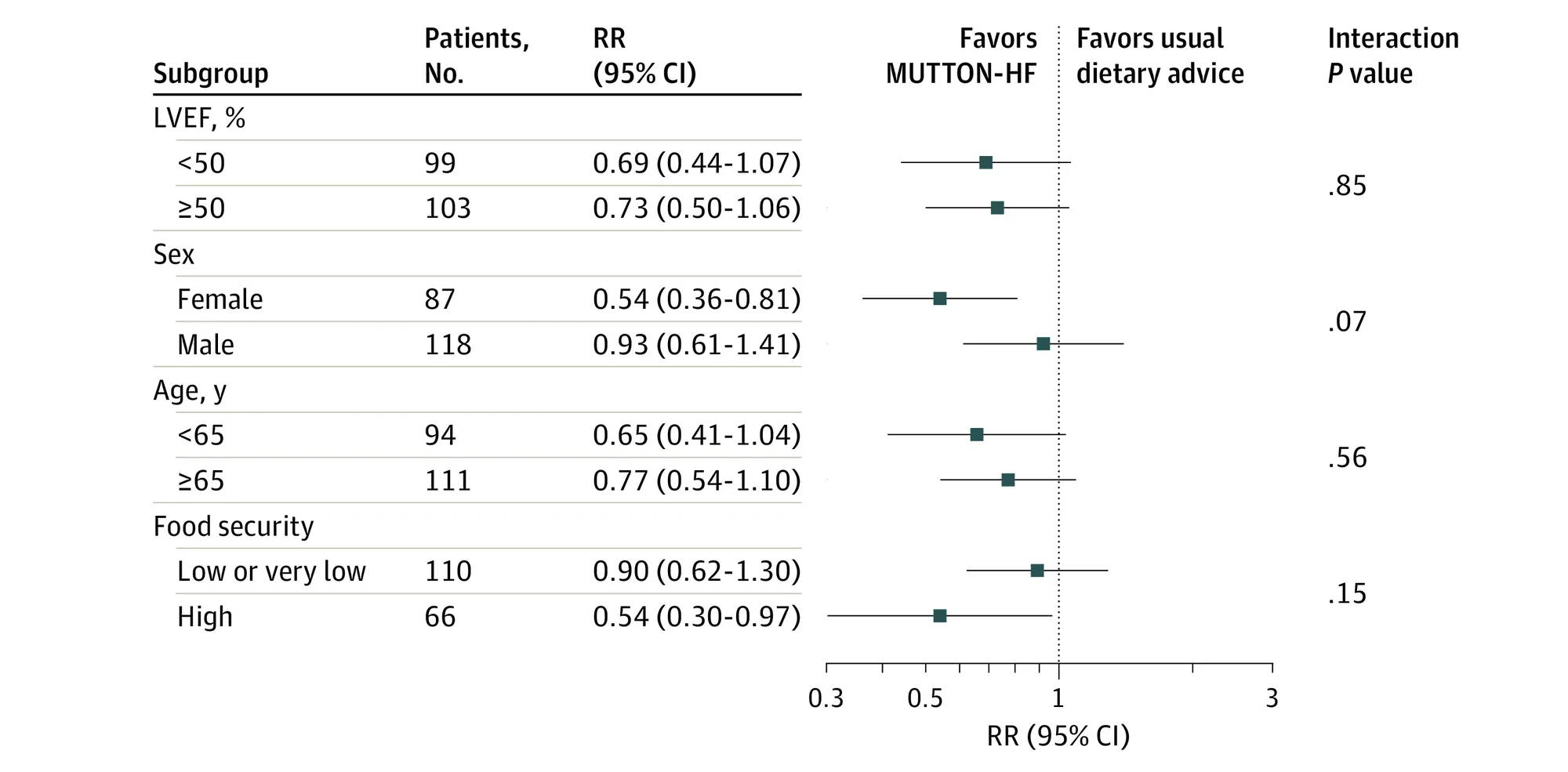
Dot Plot of Primary Outcome According to Prespecified Subgroups LVEF indicates left ventricular ejection fraction; RR, relative risk.

Total counts for events for hospitalizations and ED visits and HF-specific encounters are summarized in eTable 9 in Supplement 2. Changes in general health status and minutes of exercise weekly are shown in eTable 10 in Supplement 2. There were increases in the weekly amount of self-reported exercise in the intervention group compared with the control group. Prespecified subgroup analyses showed a consistent benefit of the MUTTON intervention across sex, age, LVEF, and baseline food security status (Figure 2).

## Discussion

As the first randomized clinical trial of an Indigenous Food is Medicine intervention to improve cardiovascular health outcomes to our knowledge, MUTTON-HF demonstrated that a locally and Native-sourced CMTM program incorporating traditional Navajo foods and recipes led to a lower incidence of hospitalization or ED visits among patients with HF in rural Navajo Nation. Additionally, there were reductions in HF hospitalizations, food insecurity, financial strain, weight, and blood pressure, as well as improvements in the KCCQ summary score and social limitation and physical limitation components.

Other studies of MTM programs in HF have demonstrated an effect on clinical outcomes but have exclusively focused on the postdischarge period and urban populations.^24,27,52^ In this pragmatic trial, we found a significant benefit in patients with broader eligibility criteria (hospitalization or ED visit during the last 12 months). It is likely that the significant benefit was due in part to high underlying rates of food insecurity among the participants. Present day effects of settler colonialism include nutrition insecurity^1,2,3,4,53^ and a lack of access to electricity and water, resulting in greater reliance on ultraprocessed food and loss of dietary diversity.^5,54,55,56,57,58^ This is further compounded by forced assimilation and genocide, leading to a loss of intergenerational transfer of Indigenous knowledge. Indigenous Food is Medicine programs are one way to rectify the profound disruptions of cultural practices and traditional relationships with the natural world that have resulted from settler colonialism.^59,60,61^

Leveraging the protective assets of Native communities is paramount to advancing Indigenous health and well-being.^1^ While the benefits to weight and blood pressure are likely due to the low-sodium and low-caloric contents of the meals, we hypothesize that the program’s benefit extends beyond nutritious content. As a strength-based intervention, MUTTON-HF was designed to address interconnected Indigenous determinants of holistic health by promoting cultural connections and multidimensional nourishment through the consumption of sacred traditional foods.^23,62,63,64,65,66^ Food Is Medicine programs in other settings should consider tailoring not only for medical needs but also for the strengths of the food environment (ie, culture and local food systems) through community-engaged design.^67,68,69^

Consistent with our pilot study and prior studies in other populations, we found significant improvements in the KCCQ summary score, particularly the physical and social limitation subscores.^23,24^ This is also reflected in significant increases in weekly exercise time among intervention participants. The MUTTON-HF program may also have improved social interactions and isolation, particularly for those receiving home delivery. This intervention also led to improvements in other domains, such as reduced financial strain. Forthcoming qualitative analyses will aim to further elucidate the holistic effect of this intervention on patient-reported outcomes and well-being and explore specific program features, such as provision of appliances, incorporation of traditional ingredients, and delivery support.

This trial, in addition to demonstrating the clinical effectiveness of MUTTON-HF, also provides further insights on implementing a CMTM intervention in rural and tribal settings.^23^ Most studies evaluating MTMs or groceries have taken place in an urban setting or used home delivery by large food corporations.^70^ Food is Medicine programs in rural settings, and across tribal reservations in particular, can pose logistical challenges due to vast distances, a lack of paved roads, and infrastructure needs. Our community-designed intervention was uniquely tailored to fit the local context by (1) sourcing from local Native farmers and ranchers to advance food sovereignty, (2) curating meal plans with traditional culturally relevant recipes and foods, and (3) adopting final mile strategies, including provision of appliances and propane and delivery to miniature hubs and homes, as needed. Given unique cost considerations, economic analyses are underway to further inform more widespread adoption and scalability to other similar settings.

### Limitations

This study had several limitations. The results may not be generalizable to other settings, particularly those with lower rates of food insecurity. While the specificity of the intervention might have also limited generalizability, core components, such as locally sourcing meals, prioritizing culturally tailored recipes, establishing distribution networks, and leveraging Community Health Worker partnerships, are relevant across other cultural and geographic contexts. Complete case analyses of secondary outcomes assume that data are at minimum missing at random. Per community preference, participants could opt out of laboratory evaluations and surveys yet still receive meals, meaning missingness was largely structural and driven by participant preference rather than health status, lending some support to this assumption. However, we cannot rule out differential missingness, and results for secondary outcomes should be interpreted with appropriate caution, as differences in people who completed surveys and laboratory evaluations could have introduced biases in unknown directions. While several biomarkers showed changes in the intervention arm, many changes were not statistically significant, potentially due to underpowering due to missing laboratory data. While infrequent, hospitalizations and ED visits to outside facilities were captured in the survey by self-reported events, which were adjudicated by obtaining and reviewing outside records. However, there could be reporting biases. The intervention, although effective, was short compared with other MTM programs in other populations and settings.^70,71,72,73^ Additional research is needed to understand the residual benefits of the intervention longer term to inform decisions on optimal duration. Without another intervention arm offering nonculturally tailored MTMs, we were unable to determine the degree to which including Indigenous foods contributed to positive outcomes vs the provision of healthy food alone. Given the high rates of nutrition insecurity in the study population, it is possible that any intervention offering nutritional support might confer substantial health benefits. However, this intervention was designed with community input to maximize acceptability, uptake, and engagement.^23^ Community-designed programs such as MUTTON-HF may advance health while also empowering tribal communities; forthcoming research will evaluate the effect of MUTTON-HF on strengthening local food systems and advancing food sovereignty.

## Conclusions

This randomized clinical trial of a community-designed CMTM program that incorporated locally sourced traditional Diné meals led to a reduction in hospitalization or ED visits within 90 days and improved food insecurity, KCCQ summary scores and physical and social limitation subscores, financial strain, weight, and blood pressure. These results provide valuable insights into the implementation and effectiveness of this Food is Medicine program in a rural tribal setting and support its expansion to other similar settings.

## Notes

Supplement 1. Trial protocol

Supplement 2. eFigure. Overview of the MUTTON-HF Intervention eTable 1. Sample Menu for 1 week of CMTM Intervention eTable 2. Nutrient Fact Panels for a Sample of MUTTON-HF Meals eFigure 2. Map of Patient Communities, Study Sites and Food Program Sites eTable 3. Additional Baseline Characteristics by Treatment Arm eTable 4. Baseline characteristics between those with complete vs. missing clinical biomarkers eTable 5. Baseline characteristics between those with complete vs. missing survey data eTable 6. Implementation Characteristics Among those in the Intervention Arm eTable 7. Distributed household appliances by group assignment eTable 8. Adverse Events by Assigned Treatment Arm eTable 9. Total Events and Outcome Counts by Assigned Treatment Arm eTable 10. Exploratory Outcomes

Supplement 3. Data sharing statement
