# Supplementary material for: An Indigenous Food Is Medicine Intervention: The MUTTON-HF Randomized Clinical Trial
Source: JAMA Intern Med. 2026 Jul 27:e262879. Online ahead of print. doi: 10.1001/jamainternmed.2026.2879 (PMC13409115; doi:10.1001/jamainternmed.2026.2879)
Supplement: Supplement 1. — Trial protocol [file jamainternmed-e262879-s001.pdf]

PROTOCOL TITLE: MUTTON-HF (Medically **U**tilized **T**ailored **T**raditional food to **O**ptimize **N**utrition) in Heart Failure

Version Date 4/30/2025

Clinicaltrials.gov Registration: NCT06549699

SECTION 1: RESEARCH PLAN

Statement of Purpose:

The American Indian and Alaska Native population has experienced significant cardiovascular health disparities compared with other racial and ethnic groups in the U.S. [1] Heart failure, in particular, causes significant morbidity and mortality in Navajo Nation. For many Navajo patients, similar to other American Indian populations, food insecurity is a major driver of health disparities. [2][3] In fact, qualitative data from our heart failure patient advisory committee have found that 89% of patients with heart failure believe nutrition insecurity is a major barrier to optimal health. Dietary factors are believed to be an important cause of hospitalizations in patients with heart failure and cardiovascular outcomes. There is increasing evidence that direct dietary support, such as produce prescription or provision of medically tailored meals may improve cardiovascular outcomes and disease-specific quality of life.[4][5] Furthermore, there has been an increased focus in Indigenous communities to reclaim traditional indigenous foods to improve health. However, more evidence of the benefit of traditional Indigenous foods for cardiovascular health is needed.

We, therefore, in discussion with community members and tribal partners at two Indian Health Service (IHS) sites in Navajo Nation, will implement and evaluate the effectiveness of a medically and Native-sourced culturally tailored meal delivery program to improve outcomes in heart failure in rural Navajo Nation. This study includes two phases, with a phase I pilot feasibility study, followed by phase II—a comparativeness effectiveness randomized controlled trial to compare the implementation of our medically and culturally tailored meal delivery program compared to usual care. Phase I outcomes included implementation outcomes such as feasibility and acceptability to inform phase II. The primary outcome for the trial in Phase II will be the proportion of patients who have a hospitalization or ER visit for any cause within 90 days post implementation period start date. Secondary outcomes will include: heart failure hospitalizations specifically, ER visits for volume overload specifically (ER visits for lower extremity edema, dyspnea, with clinical evaluation consistent with volume overload) within 90 days, Kansas City Cardiomyopathy Questionnaire clinical summary score at baseline and at 60 days; prescription adherence rates (% of filled prescriptions out of prescriptions made) for all medication as well as GDMT specifically at 60 days, weight at baseline (and BMI) at 60 days, and the following lab parameters and biomarkers at baseline and at 60 days: albumin, prealbumin, creatinine, NT-proBNP,

HbA1c, total cholesterol, LDL-C, HDL-C, Triglycerides, CRP, urine albumin:creatinine ratio. We will also collect measures of and measures of food insecurity, financial stress, Indigenous cultural connectiveness using the validated Cultural Connectedness Scale-California (CCS-CA), the Indigenous Nourishment Scale, as well as qualitative analyses of patients and local food suppliers to evaluate how this program strengthens local food systems. We will also evaluate food diaries and perform a survey at 90 days to evaluate for any sustainable change in diet and behavior post intervention. Semi-structured interviews will be performed of stakeholders including a subset of patients, primary care providers and food suppliers to assess implementation outcomes.

Probable Duration of Project: 24 months

Research Plan: We plan to conduct a randomized, comparativeness effectiveness design trial, with 1:1 randomization. This will be done at two IHS clinical sites in the Gallup Service Unit at Indian Health Service: Gallup Indian Medical Center (GIMC) and Tohatchi Health Clinic (THC), with the plan to expand to Fort Defiance Medical Center and/or Shiprock Medical Center if we are unable to enroll our target sample size.

Study Population: All patients 18 years of age or older, with diagnosis of heart failure based on ICD code I50* with a hospitalization (any cause) in the last 12 months. Only patients with active prescription through IHS and engaged in care at our centers (clinical visit within last 12 months) will be included. We will exclude patients on hospice care or those residing in an acute care or skilled nursing facility, or any patient currently residing outside of the 50-mile IHS Gallup Service Unit catchment area.

Subject Recruitment: Eligible patients are identified through a query through iCARE, a EHR based data system through the IHS EHR. Patients with ICD10 diagnostic criteria for HF will be identified (ICDI50*). The inclusion criteria will be all adults ≥18 years, who have a prescription in the IHS system in the last 12 months and have had a clinical visit at GIMC or THC in the last 12 months, as well as a hospitalization (all-cause) in the last 12 months. Patients will be consented verbally either at the health center or verbally over the phone to enroll. If we have not met our 50% enrollment goal by 6 months, we will expand enrollment to another Navajo IHS site—Fort Defiance and/or Shiprock Medical Center, and enroll patients with ER visit (rather than just hospitalization) in the last 12 months.

Enrollment: All patients that meet eligibility criteria and consent to participate will be enrolled to receive meal delivery simultaneously: all enrolled patients will start receiving the intervention at the same starting time point, t=0. Patients randomized to usual care will also start the "intervention" of usual care at the time t=0, so that all patients will have outcome assessment on the same time schedule. For operation and logistical considerations, all baseline assessment (including consent, enrollment, baseline surveys and lab work) will occur in the enrollment period (-45 to 0 days prior to the intervention period). No clinical events will be included during the enrollment period as an outcome.

Randomization: Randomization in this study will occur at the level of the patient in a 1:1 fashion. Randomization will be stratified by sex, age (<65 years, ≥65 years) and by left ventricular ejection fraction <50% versus ≥50%. Patients living in the same household will be randomized to the same treatment assignment to avoid contamination.

Intervention

- Phase I:

1. A. ***Designing Culturally Relevant Medically Tailored Meals***

We partnered with Tocabe, an Indigenous-run meal program, offering healthier and traditional foods, utilizing food from Native farmers and food producers.[6] Tocabe is a native-sourced and native-owned organization, which provides ready-made medically tailored meals to customers. In addition, they have extensive experience providing Indigenous and locally sourced foods direct to tribes. As part of Phase I and Phase II planning, we had local experts in Diné cuisine meet with our partners at Tocabe, to help develop a menu of culturally relevant meals and recipes. As part of this process, we determined key high priority Navajo food items, e.g. mutton, and recipes that are culturally important to include as part of our medically tailored meals. We designed different meal plans with a variety of options, all based on culturally relevant foods and recipes which will be incorporated as part of our medically tailored plan. Meals were designed to incorporate local and traditional Navajo foods. Meals were medically tailored to be consistent with sodium-restricted Dietary Approaches to Stop Hypertension as per American Heart Association recommendations.[6][7] Meals will be designed with cultural diet experts as well as registered dieticians to ensure meals are optimized medically and culturally. As much is feasible, we partnered with local Navajo farmers coalitions to locally source all ingredients and produce to strengthen local food systems and food sovereignty, and also to increase to increase accessibility of the meals and the likelihood of long-term adoption of recipes and meals after the intervention period.

1.B ***Supply Chain and Meal Delivery Logistics***

As part of Phase I, we established and secured a supply chain for meals, and secured logistics for meal delivery, particularly for those living rurally on the reservation. Among a sample of n=22 HF patients, we found that 95% of patients had PO boxes (without a physical address/mailbox). Given this, we have partnered with the Gallup Food Pantry, and will have frozen meals prepared, packaged, and delivered to the Gallup Food Pantry in bulk as a central food hub, with subsequent patient distribution. Patients that live locally and are able to pick up meals will come pick up meals weekly. Those who are unable to pick up meals (or do not come for their meals), will have meals delivered weekly through various mechanisms (per patient preference) that were tested and deemed successful in our pilot trial. These include delivery to a minihub (Chapter House, Tohatchi Health Clinic) for pick-up, delivery by community health representative (CHR) or nurse, and peer delivery (pick up at food pantry and delivery by another participant living in same community for peer that is unable to pick up). For those without refrigeration, meals will be delivered to local chapter houses, and patients will pick up 3-day supply of meals in freezer boxes. In addition to refrigeration, we will also assess and address household infrastructure (e.g. ability to store and heat meals). We developed these logistical plans and evaluated them in our pilot feasibility trial, which has informed operationalizing our phase II comparativeness effectiveness trial.

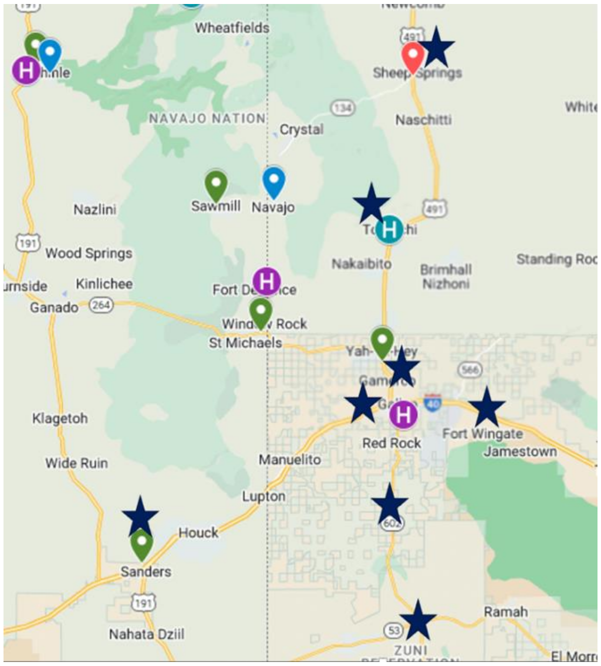

1. C. Pilot Feasibility Trial

Prior to trial enrollment, we conducted a 6-month feasibility trial in which we enrolled a total of 20 patients, to test feasibility of home delivery of meals and obtain patient input on acceptability to optimize our larger scale trial. Our IHS study facilities have a catchment area of over a 50-mile radius. Of 20 patients enrolled, patient's locations of residence are shown in the map.
Given the geographic distance between patients, the rurality of locations, and lack of mailboxes or mailing addresses for many patients, we conducted a pilot feasibility study of 20 patients to ensure feasibility of patient delivery and acceptability of the medically and culturally tailored meals. Patients received 2 meals daily (14 meals weekly) for 4 weeks. Meals were prepared, packaged, and delivered in bulk to our central food hub (Gallup Food Pantry). From there, based on patient preference, a 1-week supply of meals was either picked up weekly by the patient or delivered by preferred patient mode of delivery (delivery to minihub, delivery by CHR, peer delivery). Patients also received 1 cooking class to learn how to make recipes from the meal plan, all incorporating locally available ingredients.

We assessed implementation outcomes including feasibility, acceptability, adoption, and fidelity of the intervention. Complete details on the pilot feasibility study protocol and outcomes are available online.[8][9]. Given implementation success as defined, we will proceed with phase II, with study design and outcomes informed by results of our pilot trial.[9]

- Phase II: Comparativeness effectiveness trial of Medically and traditionally tailored meals for Heart Failure

We will conduct a comparativeness effectiveness trial to evaluate the impact of providing medically and culturally tailored Indigenous focused meals delivered to heart failure patients. Meals will be prepared, packaged, and delivered once weekly, with delivery mechanism based on community and patient preference. Two meals will be provided daily for 60 days. Along with the meals, patients will also receive recipe cards with each meal, as well as educational information regarding the meals, traditional food items included, and nutritional information, to educate on the health benefits of the food provided, as well some information regarding the traditional foods included and from where they were sourced. Additionally, patients will receive one cooking class conducted by a Diné Registered Dietician (Denee Bex) to learn how to make a few key recipes from the meal plan, all utilizing locally available ingredients.

Baseline Characteristics: In addition to the American Heart Association sociodemographic core measures, we will also collect tribal affiliation, language, and in which local chapter/community patients reside.

<u>Endpoints:</u> The primary outcome for the trial will be the proportion of patients who have a hospitalization or ER visit for any cause within 90 days post intervention period (all patients will start the study at t=0 as described above). We will not include hospitalizations that occur electively for an elective procedure.

Secondary outcomes will include: heart failure hospitalizations specifically, ER visits for volume overload specifically (ER visits for lower extremity edema, dyspnea, with clinical evaluation consistent with volume overload), change in weight and body mass index at 60 days from baseline, Kansas City

Cardiomyopathy Score (and its components including summary score, physical limitation score, symptom frequency score, quality of life score and social limitation score)[10], and the following lab parameters and biomarkers at baseline and at 60 days: albumin, prealbumin, creatinine, NT-proBNP, HbA1c, Total cholesterol, LDL-C, HDL-C, Triglycerides, CRP, urine albumin:creatinine ratio. Lab work will be permitted to be collected 14 days before 60 days mark and up to 30 days after (from 46 days-90 days post intervention period).

Additional secondary outcome measures:

Diet Quality

- We will assess diet quality using the 10-item DSQ [11] with an addition question to assess traditional Diné food intake (*During the past month, how often did you eat traditional Diné foods (such as blue corn mush, steamed, roasted or dried corn, sumac berries, mutton, local varieties of squash or beans)* at baseline and at the end of the intervention period.

Food Security

- We will evaluate baseline and 60-day rates of food insecurity utilizing the USDA Adult Food Security: Six Item short Form survey (USDA FSSM 6-Item).[12] At the 60-day evaluation, we will adapt the USDA FSSM 6-Item questionnaire to evaluate food insecurity within the last 2 (rather than 12 months).

Indigenous Cultural Connectedness:

- We will measure cultural connectedness utilizing a 6-question modified version of the validated Cultural Connectedness Scale-California (CCS-CA) at baseline and at 60 days.[13] This is a validated score, developed by Indigenous scholars to measure cultural connectiveness in Indigenous populations, and has been shown to correlate with physical health and be a social determinant of health.[14] This scale has previously been modified for use in Diné populations specifically by our group.

Indigenous Nourishment Scale

- We will evaluate the Indigenous Nourishment Scale (Measure A) [15], which is a scale developed through community-based methods in Indigenous communities to encompass multiple dimensions of nourishment. This scale offers an Indigenous framework to allow for health measurements rooted in Indigenous perspectives. We will evaluate Measure A of this scale at baseline and 60-days. We will score each question from 0 (never) to 6 (always) for a total score 0-54.

Financial Stress

- We will measure Financial Stress Scale at baseline and at 60 days using two questions:[16]

- How worried were you over the last 2 months about paying for housing (rent, mortgage, etc.)?
- How worried were you over the last 2 months about paying monthly bills?
    - All questions will be answered on a 4-point scale, where 1 = “not worried at all”, 2 = “not too worried”, 3 = “moderately worried”, and 4 = “very worried”

Intervention characteristics:

- We will evaluate the following in partnership with Tocabe:
    - % of meals that include locally sourced ingredients
    - % of food supply sourced by Diné producers/suppliers/farmers
    - % of meals sourced by Native suppliers/farmers

Implementation Outcomes: Fidelity, acceptability, feasibility, adoption:

- We will evaluate the percentage of meals delivered successfully to the patient (or percentage of meals that were successfully picked up by the patient if picked up from the food pantry or other location).
- We will assess baseline dietary patterns through 3-day food diaries during week 1 and week 8 after enrollment. Additionally at 90 days, we will perform a survey to evaluate dietary patterns post intervention and adoption of any of the recipes/behavioral changes.
- We will evaluate patient satisfaction by evaluating the Net Promoter Score (i.e. how likely is it that you would recommend this program to a community member?”) post-intervention.
- We will evaluate fidelity and compliance with survey at the end of the program to assess how many of the meals were consumed by the patient.
- We will perform semi-structured interviews of non-patient stakeholders (i.e. farmers that supplied produce, livestock farmers, food pantry) to assess acceptability and feasibility of providing produce for the meals, as well as the degree to which the program contributed to strengthening the local food system. We will also perform semi-structured interviews of primary care providers and a subset of patients. Semi-structured interviews will be guided by the Consolidated Framework for Implementation Research (CFIR), and we will explore multiple constructs within each CFIR domain that are hypothesized by the study team and based on existing literature to be relevant to acceptability and feasibility of the program.[17]

Exploratory Outcomes:

Additional exploratory outcomes will include time to first hospitalization and ER visit post-intervention period, prescription adherence rates (defined as percentage of filled prescriptions out of total prescriptions) for all medication as well as guideline-directed medical therapy specifically (for heart failure with reduced ejection fraction this would include ACEi/ARB/ARNI, Beta-blocker, SGLT2 inhibitors, and mineralocorticoid receptor antagonist; for HFpEF this would include SGLT2 inhibitors). In addition to 60-day measurements, we will also collect weight measurements at 30 days and 90 days.

Physical Activity

- We will evaluate how much physical activity/exercise patients are participating in weekly at baseline, and at 60 days by asking patients to estimate the number of minutes weekly that they are participating in formal exercise or physical activity.

General Health Status:

- We will evaluate patients' general health status using a single general health status question (would you say that in general your health is excellent, very good, good, fair, or poor) at baseline and post-intervention.

Outcomes determined by review of medical, hospital, and billing records, as well as patient surveys and semi-structured interviews. We will also extract medication at baseline and at 60 days to assess for changes in medical therapy. All patients will be provided a scale and blood pressure cuff for weight and blood pressure measurements, respectively. Patients will be instructed to take weights first thing in the morning (or pick a time-up front for all weight measurements for consistency). All patient-reported measures, including KCCQs and CCS-CA scores will be over the phone or in person per patient preference. Patients will come in for lab work at baseline and at 60 days. For patients who are unable to come with for lab work at 60 days (and within the window of 45-90 days), then we will have public health nurses provide outreach to collect blood work in the home. Patients will receive a text message (if they have a cell phone) or phone call on the landline (if no cell phone) to remind them to complete food diaries, weights, and other patient-reported measures in a timely manner. If patients are unable to come in for baseline lab work prior to intervention period, we will use completed lab work as baseline lab work if within 90 days 90 days of the intervention period. For those that have trouble completing written food diaries, we will collect this information verbally over the phone.

For subsequent planning purposes, we will also track costing data to determine the complete cost of the intervention and estimate total program costs (including all resources, even donated, involved in the implementation of the program, but not including research activities such as blood tests).

<u>Comparator</u>: Those randomized to usual care will not receive any home delivery meals but will receive the recipes of healthy recipes that incorporate traditional local foods and educational booklet. They will also receive a gift bag for agreeing to participate in the study and a $50 gift card upon completion of the study. Given ethical concerns about those not enrolled to receive the food is medicine intervention, after discussion with our community partners, we will provide 2 meals daily (14 meals weekly) for 4 weeks for all patients randomized to the control group after the study period (and outcome assessment) is complete if patients wish to receive meals and no safety concerns are identified.

<u>Blinding of Intervention:</u> Patients will be informed that they are enrolled in a program to receive medically and culturally tailored meals at home, and thus will not be blinded to their randomization status or participation in this trial. Providers will be not blinded to the intervention. Outcomes will be pulled from the EHR from study members, and those performing the analyses will be blinded to randomization status of the patient.

<u>Enrollment Period:</u> To allow time to coordinate the necessary operational and logistical infrastructure for meal delivery based on patient location/community and delivery mechanism, patients will be

consented and enrolled t=-45 to t=-1 days prior to the start of the intervention period (t=0 for all patients). During this time, baseline assessment with surveys and lab work will take place. All patients will then start the study period (and outcome assessment period) at the same t=o. Any events that take place in the -45 to -1 days prior to the intervention period will not be included as an outcome. Summary of enrollment, intervention, and outcome assessment timeline is shown below.

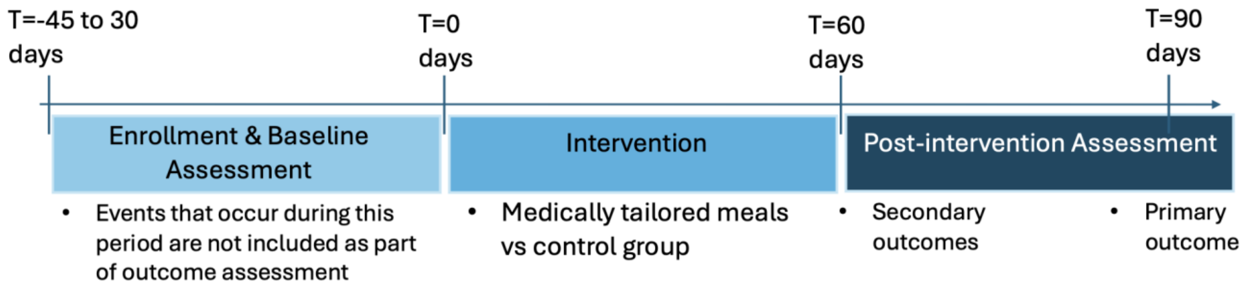

<u>Intervention Duration</u>: The study team will implement the food intervention for 60 days.

<u>Inclusion/Exclusion Criteria</u>

Inclusion

- Age >=18 years
- ICD I50* diagnosis
- Clinical encounter in last 12 months
- Prescription in IHS system in past 12 months
- Primary care physician at one of the two IHS sites
- Hospitalization or ER visit (any cause) in the past 12 months

Exclusion

- Hospice care
- Living in acute rehabilitation or skilled nursing facility
- Living outside the Gallup Service Unit (outside 50-mile catchment area)

How will eligibility be determined, and by whom?

- Eligibility of patients will be assessed by the study team based on the patient’s medical record.

Methods to Reduce Loss to Follow-up: Patients that do not pick up meals in the planned mechanism within 2 days of planned pick up, will have meals delivered to them at home by a CHR. If patients are not participating in the study as planned, they will be contacted by phone. If unable to reach by phone x 3 attempts, family members will be contacted. If unable to reach anyone, then we will have a public health nurse/community health representative conduct a home visit.

Data Collection Timeline

Time Period	Baseline	Intervention 30 days	Intervention 60 days	60 days	90 days
Patient Surveys/<br>Patient Related Outcomes					
	X			X	
	- KCCQ12<br>- USDA Food insecurity<br>- DSQ10<br>- CCS Score<br>-Nourishment Scale<br>- Financial Stress Scale<br>- Exercise<br>– Health status			- KCCQ12<br>- USDA Food insecurity<br>- DSQ10<br>- CCS Score<br>-Nourishment Scale<br>- Financial Stress Scale<br>- Exercise<br>– Health status	
Lab work					
	X			X	
	- CMP<br>- CRP<br>- HbA1c<br>- Lipid panel<br>- Prealbumin			- CMP<br>- CRP<br>- HbA1c<br>- Lipid panel<br>- Prealbumin	
Clinical measures					
	X	X		X	X
	Blood Pressure			Blood Pressure	
	Weight	Weight		Weight	Weight
Primary Outcome: ER visits and hospitalizations					
	X	X		X	X (90 days)
Food Survey					X

In-Kind Funding

- **The Indian Health Service** – The IHS will fund all patient scales, allow for lab work to occur at IHS facilities free of charge, and provide funding for patient navigators and heart failure nurses to assist in tracking patients, calling patients to remind them of lab work, assisting with completion of patient-reported outcomes/surveys and case report forms for trial outcomes. They will also provide a freezer at Tohatchi Health Clinic (to serve as a mini-hub for Northern catchment area).
- **Dr. Eberly** with funding through the **Robert A. Winn Diversity in Clinical Trial Career Development Award** will fund clinical trial support through the clinical research collaboration unit at Penn.
- **Tocabe Inc**- will donate recipe cards of the meals provided, as well as the recipe cards for the control group.
- **The Gallup Food Pantry-** will donate their freezer space and have agreed to serve as the central food hub to meal delivery and will provide their services for decentralized patient meal delivery including their mobile delivery unit as needed.

Additional Stakeholders and Partners

- **Navajo Area Department of Health**- Dr. Paula Mora, who serves as the Director for the Navajo Area Department of Health for the IHS, is fully supportive of this study and will serve on our community advisory board. She works closely with the Navajo Nation Department of Health, who are also supportive of this work. We will be working closely with the Navajo Nation Department of Health on this study so that if our program is effective, we can leverage our partnerships with tribal leaders to secure pathways to allow for sustainable funding and adoption of this program long-term.
- **Indian Health Service Headquarters Office of Quality:** Our team includes Dr. Benjamin Feliciano, improvement advisor, and Dr. Philippe Champagne, director of quality improvement for the IHS HQ Office of Quality Division of Innovation. This study is supported by an Indian Health Service Innovations Award, which provides logistical support from the IHS Office of Quality. Additionally, as part of an IHS innovations award, the results of this study may inform adoption and expansion more broadly throughout the IHS of such programs.
- **Indian Health Service Headquarters Food is Medicine Coalition:** Our community advisory board includes Dr. Stacy Hammer who is the principal nutrition consult for the IHS nationally and serves as the IHS representative for the federal agencies' Food is Medicine Coalition. These results will be critical to allow the coalition to advocate for increased congressional funding to support food is medicine initiatives throughout the HIS nationally.
- **Shiprock Farmers Coalition/Local Navajo Farmers:** To locally source ingredients for our meals with Tocabe, we will be partnering with local farmers coalitions such as the Shiprock Farmers Coalition and other local produce and livestock farmers to support local economic development and support local food systems.

Community Advisory Board

We have established a community advisory board of Our community advisory board will include the following individuals to ensure not only success of the proposed research project, but also to allow for sustainability of the program if found to be effective:

- Dr. Paula Mora, the Director of Navajo Area Department of Health
- Stacy Hammer, RD- Principal nutrition consult for the IHS Headquarters, IHS representative for the federal agencies' Food is Medicine Coalition
- Sharon Sandman- Community-based partner leading initiatives to support local farmers and food sovereignty throughout Navajo Nation
- Colleen Biakeddy - Diné (Navajo) sheep rancher, expert in traditional Navajo sheep herding. She will help locally source USDA approved Mutton for meal delivery and to support local livestock farmers as well as ensure traditional and ethical use of sheep for meals, as well as provide expertise on experience of challenges facing local farmers to source produce and meat.
- Commander Pamela Detsoi-Smiley: Chief Executive Officer of Gallup Indian Medical Center of the Indian Health Service
- Rory Aufderheide-Primary care physician at one of the IHS sites (THC), on the Board of Directors for Gallup Food Pantry with expertise in food is medicine initiatives locally.
- Jacob Manche- Director of Quality, Gallup Indian Medical Center, Indian Health Service
- Duane 'Chili' Yazzie- Diné (Navajo) farmer and Earth Defender; Director of ToohBAA farming coalition, advocate for Indigenous human rights, Navajo food sovereignty, and fighting exploitation of our Earth Mother

- Patient and caregiver- we will invite at least 1-2 patients and caregivers from our previously implemented patient advisory heart failure board to join our community advisory board for this study.

We will meet monthly with the community advisory board during the planning stages, and then bimonthly during implementation to center community perspectives and assess implementation outcomes and feedback, followed by quarterly meetings after the active implementation phases.

Study Timeline

	YEAR 1												YEAR 2											
	M1	M2	M3	M4	M5	M6	M7	M8	M9	M10	M11	M12	M13	M14	M15	M16	M17	M18	M19	M20	M21	M22	M23	M24
Phase I: Meal Curation of Traditional Navajo Meals	X	X	X																					
Phase I: Secure supply chain	X	X	X																					
Phase I: Secure local suppliers for produce and livestock		X	X	X																				
Eastern Navajo Agency Council Approval			X	X	X																			
IRB Approval				X	X																			
Phase I: Pilot feasibility (n=20)					X	X																		
Phase I: Pilot feasibility outcome assessment						X	X																	
Phase II: Trial																								
Determine eligible sample in our healthsystem		X	X	X	X	X	X																	
Enrollment								X	X															
25% Sample Size (n=50)									X	X														
50% Sample Size (n=100)										X	X													
75% Sample Size (n=150)											X	X												
100% Sample Size (n=200)												X	X											
Study Period-Primary Outcome																X								
Study Period-ALL Outcome Assessments Ends																			X					
Data Analyses																			X	X	X			
Present to Stakeholders and Tribal Council																					X	X	X	
Dissemination																						X	X	X

Protection of Human Subjects: Protection of Human Subjects:

This study is aimed at implementing a model to improve access to healthy, nutritious foods for patients. Therefore, this poses a minimal risk to patients. There is prior literature that suggests that medically tailored meal delivery improves quality of life, including Kansas City Cardiomyopathy Questionnaire summary score, as well as potentially a trend to lower heart failure hospitalizations, without significant diet-related adverse events.[4]

However, there are no prior studies of medically and culturally tailored meals in this population. However, our pilot feasibility trial has demonstrated no safety concerns and we will be medically tailoring meals to meet guideline recommendations.[6][7] Meals will be designed with registered dietician input to ensure safety is optimized. Given the short duration of the intervention and low-risk of the intervention, we will not perform interim safety analyses.

<u>Human subjects' involvement, characteristics, and design</u>: The studies outlined in this proposal depend on the enrollment of individuals with heart failure. No vulnerable populations are being specifically

targeted. We are limiting enrollment to individuals above age 18 years as the etiology and practices surrounding heart failure in pediatrics populations differ significantly from those in adults. All data is transmitted in encrypted and secure fashion, stored on servers with "triple-lock" certification, and is available only to members of the study team, IRB, and any state or federal agencies with auditing power.

Sources of Materials: No biological materials will be obtained or stored as part of these studies. Only data, as collected during set time points from the EHR will be obtained. Data includes medical record elements such as demographics, pharmacy records such as medication prescription and dosing, laboratory values, and administrative codes. All data will be stored without PHI. However, we will retain a linking dataset to be able to re-link individual data to actual patients for future studies and ongoing efforts through the HIS. Access to individually identifiable information will be limited to the PI of the study, and only then via a linking file as aforementioned. All data used for analysis and dissemination to other investigators will be de-identified.

Over or under treatment: Providers will be blinded to patient treatment assignment and thus it is unlikely that treatment will differ between patients. However, we will also be extracting medication changes during the study period to ensure there were no significant differences in medical therapy treatment between the two groups.

Potential benefits of the proposed research to the subjects and others: Subjects in this study may directly benefit from receiving healthier food options for their care. In addition, this may improve their knowledge on healthy and traditional foods and dietary practices that may be sustained after the duration of the study. Additionally, the results of these studies may lead to significant benefit in the IHS and other rural Indigenous communities. This model could be similarly expanded to other sites. The risk/benefit ratio, given the minimal risk to study subjects, is more than acceptable in this series of studies.

Data and Safety Monitoring Plan:

• What is the investigator’s assessment of the overall risk level for subjects participating in this study?

We believe that this poses minimal risk to the patients.

• If children are involved, what is the investigator’s assessment of the overall risk level for the children participating in this study? No children will be involved.

This study poses **i. Minimal risk** ii. Greater than minimal

The principal investigators (PI) is responsible for monitoring the data, assuring protocol compliance, and conducting the safety reviews at the specified frequency regularly. During the review process the PIs will evaluate whether the study should continue unchanged, require modification/amendment, or close to enrollment. The PIs or the Navajo Nation Human Research Review Board (Navajo Nation IRB) have the authority to stop or suspend the study or require modifications.

This protocol presents minimal risks to the subjects and Unanticipated Problems Involving Risks to Subjects or Others (UPIRSOs), including adverse events, are not anticipated. In the unlikely event that

such events occur, Reportable Events (which are events that are serious or life-threatening and unanticipated (or anticipated but occurring with a greater frequency than expected) and possibly, probably, or definitely related) or Unanticipated Problems Involving Risks to Subjects or Others that may require a temporary or permanent interruption of study activities will be reported immediately (if possible), followed by a written report within 5 calendar days of the Principal Investigator becoming aware of the event to the IRB (using the appropriate forms from the website) and any appropriate funding and regulatory agencies. The investigator will apprise fellow investigators and study personnel of all UPIRSOs and adverse events that occur during the conduct of this research project through regular study meetings and via email as they are reviewed by the principal investigator.

Statistical Considerations

Sample Size Determination: The primary outcome is the rate of hospitalization or ER visits within 90 days post randomization. Our preliminary data suggests that the hospitalization rate and ER visit for 90 days is approximately 29%, and based on prior data, we hypothesize a reduction to 13% [4]. We will therefore need a sample size of 204 patients to detect a statistically significant difference with 80% power and an alpha error rate of 0.05.

Interim Analysis: Given no safety concerns during our pilot trial, the short duration of the intervention, and low-risk of the intervention, we will not perform interim safety analyses.

Statistical Analysis:

The primary analysis will use the intention-to-treat principle. Between-group differences in baseline characteristics will be assessed using χ2 tests for dichotomous variables and 2-sample t tests for continuous variables. Within-group randomization to week 8 changes in continuous outcomes including laboratory evaluation and biomarkers, CCS, and KCCQ score, will be evaluated using paired t-test.

Per-Protocol Analysis and Compliance Adjustment Analysis

We will also incorporate a per-protocol analysis to assess the impact of adherence on study outcomes, ensuring a robust evaluation of the intervention's effectiveness beyond intention-to-treat (ITT) analysis.

Per-Protocol Analysis Strategy: Participants will be included in the per-protocol cohort if they meet the following compliance criteria:

- ≥70% adherence to meal delivery (defined as receiving and consuming at least 42 of the 60 intervention meals).

- Completion of baseline and 60-day assessments and lab parameters.

- The per-protocol population will be analyzed separately to determine if high adherence to the medically tailored meal intervention is associated with improved clinical outcomes compared to the usual care group.

- Results will be compared to the intention-to-treat (ITT) analysis to identify potential differences in effect size due to adherence variability.

Compliance Adjustment Analysis:

To address differential adherence, the following statistical approaches will be applied:

1. Inverse Probability Weighting (IPW):

• Weights will be assigned based on baseline covariates associated with adherence to adjust for differences between adherent and non-adherent participants.

2. Instrumental Variable (IV) Approach:

• If adherence is influenced by external factors (e.g., household food availability), an IV approach will be explored to estimate the causal effect of the intervention under full compliance conditions.

3. Multiple Imputation for Missing Compliance Data:

• Missing adherence data will be handled using multiple imputation techniques to minimize bias and maintain analytical power.

Subgroup: We perform subgroup analysis based on EF (LVEF <50, and >50%), gender, age (<65 and >=65 years), and food insecurity (those with low or very low food security vs. food secure). We will also analyze for significant changes in weight in those with obesity (BMI ≥ 30mg/kg2), HbA1c among those with diabetes (Hba1c ≥ 7), and cholesterol (total, triglycerides and LDL) among those with baseline hyperlipidemia.

Semi structured interviews will be audio-recorded and transcribed for thematic analysis. Transcripts will be analyzed using an integrated approach, an iterative process to determine themes and patterns present in the data.[18] The interviewers will develop the initial codebook together, and then will independently dual code subsets of the transcripts to identify common themes and iteratively develop a final codebook, with periodic assessment of inter-rater reliability. We will follow the consolidated criteria for reporting qualitative research (COREQ) guidelines.[19]

References

1. Eberly LA, Shultz K, Merino M, Brueckner MY, Benally E, Tennison A, Biggs S, Hardie L, Tian Y, Nathan AS, Khatana SAM, Shea JA, Lewis E, Bukhman G, Shin S, Groeneveld PW. Cardiovascular Disease Burden and Outcomes Among American Indian and Alaska Native Medicare Beneficiaries. JAMA Netw Open. 2023 Sep 5;6(9):e2334923. doi: 10.1001/jamanetworkopen.2023.34923. PMID: 37738051; PMCID: PMC10517375.
2. Hutchinson RN, Shin S. Systematic review of health disparities for cardiovascular diseases and associated factors among American Indian and Alaska Native populations. PLoS One. 2014 Jan 15;9(1):e80973. doi: 10.1371/journal.pone.0080973. PMID: 24454685; PMCID: PMC3893081.
3. Berryhill K, Hale J, Chase B, Clark L, He J, Daley CM. Food security and diet among American Indians in the Midwest. J Community Health. 2018;43(5):901–7.
4. Hummel SL, Karmally W, Gillespie BW, Helmke S, Teruya S, Wells J, Trumble E, Jimenez O, Marolt C, Wessler JD, Cornellier ML, Maurer MS. Home-Delivered Meals Postdischarge From Heart

Failure Hospitalization. Circ Heart Fail. 2018 Aug;11(8):e004886. doi: 10.1161/CIRCHEARTFAILURE.117.004886. PMID: 30354562; PMCID: PMC6205816.

5. Hager K, Du M, Li Z, Mozaffarian D, Chui K, Shi P, Ling B, Cash SB, Folta SC, Zhang FF. Impact of Produce Prescriptions on Diet, Food Security, and Cardiometabolic Health Outcomes: A Multisite Evaluation of 9 Produce Prescription Programs in the United States. Circ Cardiovasc Qual Outcomes. 2023 Sep;16(9):e009520. doi: 10.1161/CIRCOUTCOMES.122.009520. Epub 2023 Aug 29. PMID: 37641928; PMCID: PMC10529680.
6. Eckel RH, Jakicic JM, Ard JD, Hubbard VS, de Jesus JM, Lee I-M, Lichtenstein AH, Loria CM, Millen BE, Miller NH, Nonas CA, Sacks FM, Smith SC, Svetkey LP, Wadden TW, Yanovski SZ. 2013 AHA/ACC Guideline on lifestyle management to reduce cardiovascular risk. A report of the American College of Cardiology/American Heart Association Task Force on Practice Guidelines.Circulation. 2013; 129(25 suppl 2):S76–S99. doi: 10.1161/01.cir.0000437740.48606.d1
7. Appel LJ, Frohlich ED, Hall JE, Pearson TA, Sacco RL, Seals DR, Sacks FM, Smith SC, Vafiadis DK, Van Horn LV. The importance of population-wide sodium reduction as a means to prevent cardiovascular disease and stroke: a call to action from the American Heart Association.Circulation. 2011; 123:1138–1143. doi: 10.1161/CIR.0b013e31820d0793
8. Medically Utilized Tailored Traditional food to Optimize Nutrition in Heart Failure Trial Website (Mutton-HF.com). Accessed January 5, 2025.
9. https://clinicaltrials.gov/study/NCT06675331. Accessed January 5, 2025.
10. Green CP, Porter CB, Bresnahan DR, Spertus JA. Development and evaluation of the Kansas City Cardiomyopathy Questionnaire: a new health status measure for heart failure. J Am Coll Cardiol. 2000 Apr;35(5):1245-55. doi: 10.1016/s0735-1097(00)00531-3. PMID: 10758967.
11. Dietary Screener Questionnaire (DSQ) 1- item scale. dsq-quick-guide_11-2023.pdf (nutritionincentivehub.org). Accessed June 9th 2024.
12. U.S. Household Food Security Survey Module: Six-Item Short Form Economic Research Service, USDA September 2012 Six-item Short Form Food Security Survey Module (usda.gov) Accessed 6/9/24.
13. Snowshoe A, Crooks CV, Tremblay PF, Craig WM, Hinson RE. Development of a cultural connectedness scale for first nations youth. Psychol Assess. 2015;27(1):249. https://doi.org/10.1037/a0037867.
14. Masotti P, Dennem J, Bañuelos K, Seneca C, Valerio-Leonce G, Inong CT, King J. The Culture is Prevention Project: measuring cultural connectedness and providing evidence that culture is a social determinant of health for Native Americans. BMC Public Health. 2023 Apr 21;23(1):741. doi: 10.1186/s12889-023-15587-x. PMID: 37085784; PMCID: PMC10120477.
15. Maudrie TL, Caulfield LE, Nguyen CJ, Walls ML, Haroz EE, Moore LR, Dionne-Thunder RG, Vital J, LaFloe B, Norris A, Dionne V, Pain On Hip V, Dickerson J, Hawk Lessard K, Stately AL, Blue Bird Jernigan V, O'Keefe VM. Community-Engaged Development of Strengths-Based Nutrition Measures: The Indigenous Nourishment Scales. Int J Environ Res Public Health. 2024 Nov 11;21(11):1496. doi: 10.3390/ijerph21111496. PMID: 39595763; PMCID: PMC11593815.
16. Ryu S, Fan L. The Relationship Between Financial Worries and Psychological Distress Among U.S. Adults. J Fam Econ Issues. 2023;44(1):16-33. doi: 10.1007/s10834-022-09820-9. Epub 2022 Feb 1. PMID: 35125855; PMCID: PMC8806009.

17. Damschroder LJ, Aron DC, Keith RE, Kirsh SR, Alexander JA, Lowery JC. Fostering implementation of health services research findings into practice: a consolidated framework for advancing implementation science. Implement Sci 2009;4:50.
18. C U. Grounded theory for qualitative research: A practical guide. Thousand Oaks, CA: SAGE Publications; 2013.
19. Tong A, Sainsbury P, Craig J. Consolidated criteria for reporting qualitative research (COREQ): a 32-item checklist for interviews and focus groups. Int J Qual Health Care 2007;19:349-57.
