# Supplementary material for: An Indigenous Food Is Medicine Intervention: The MUTTON-HF Randomized Clinical Trial
Source: JAMA Intern Med. 2026 Jul 27:e262879. Online ahead of print. doi: 10.1001/jamainternmed.2026.2879 (PMC13409115; doi:10.1001/jamainternmed.2026.2879)
Supplement: Supplement 2. — eFigure. Overview of the MUTTON-HF Intervention eTable 1. Sample Menu for 1 week of CMTM Intervention eTable 2. Nutrient Fact Panels for a Sample of MUTTON-HF Meals eFigure 2. Map of Patient Communities, Study Sites and Food Program Sites eTable 3. Additional Baseline Characteristics by Treatment Arm eTable 4. Baseline characteristics between those with complete vs. missing clinical biomarkers eTable 5. Baseline characteristics between those with complete vs. missing survey data eTable 6. Implementation Characteristics Among those in the Intervention Arm eTable 7. Distributed household appliances by group assignment eTable 8. Adverse Events by Assigned Treatment Arm eTable 9. Total Events and Outcome Counts by Assigned Treatment Arm eTable 10. Exploratory Outcomes [file jamainternmed-e262879-s002.pdf]

Supplemental Online Content

Eberly LA, George C, Sandman S, et al. An Indigenous Food is Medicine intervention: the MUTTON-HF randomized clinical trial. *JAMA Intern Med*. Published online July 27, 2026. doi:10.1001/jamainternmed.2026.2879

eFigure. Overview of the MUTTON-HF Intervention

eTable 1. Sample Menu for 1 week of CMTM Intervention

eTable 2. Nutrient Fact Panels for a Sample of MUTTON-HF Meals

eFigure 2. Map of Patient Communities, Study Sites and Food Program Sites

eTable 3. Additional Baseline Characteristics by Treatment Arm

eTable 4. Baseline characteristics between those with complete vs. missing clinical biomarkers

eTable 5. Baseline characteristics between those with complete vs. missing survey data

eTable 6. Implementation Characteristics Among those in the Intervention Arm

eTable 7. Distributed household appliances by group assignment

eTable 8. Adverse Events by Assigned Treatment Arm

eTable 9. Total Events and Outcome Counts by Assigned Treatment Arm

eTable 10. Exploratory Outcomes

This supplemental material has been provided by the authors to give readers additional information about their work.

© 2026 Eberly LA et al. *JAMA Internal Medicine*.

© 2026 Eberly LA et al. *JAMA Internal Medicine*.

Supplemental Figure 1. Overview of the MUTTON-HF Intervention

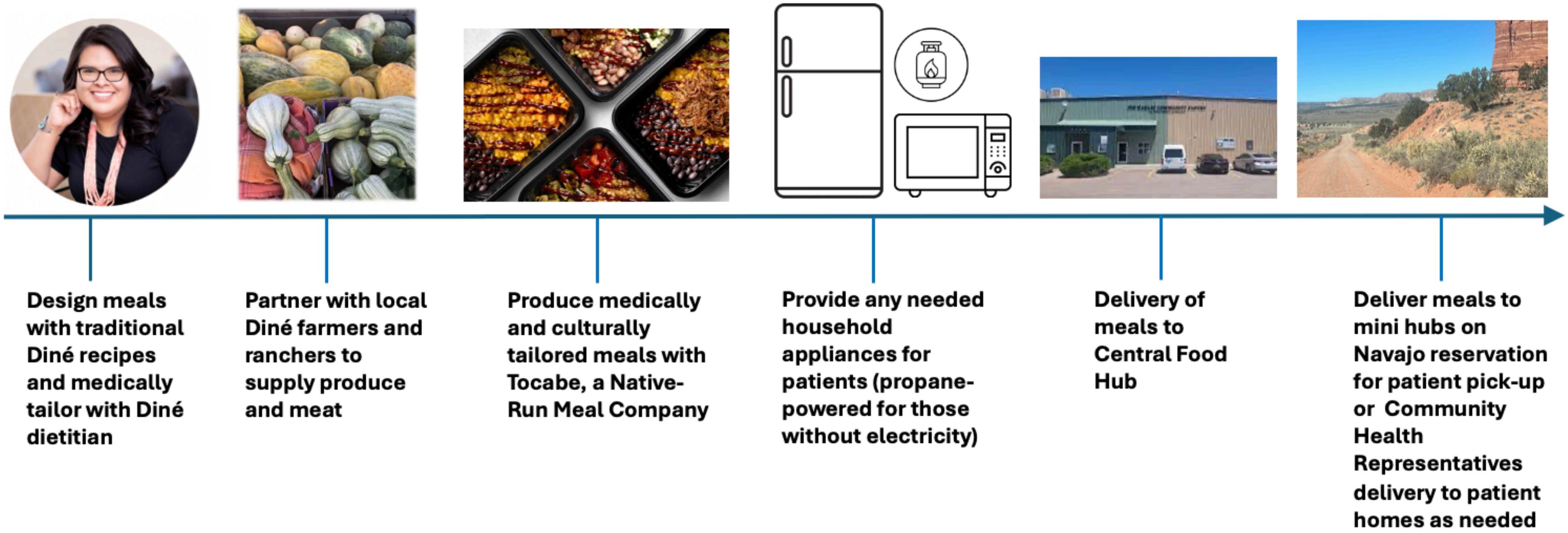

© 2026 Eberly LA et al. *JAMA Internal Medicine*.

Supplemental Table 1. Sample Menu for 1 week of CMTM Intervention

14 Weekly Meals	Sample Meal s	Ingredients	Sample Meal Photos
7 breakfasts	Chiiłchin Apple Harvest Oatmeal	Sumac berry, oatmeal, apple, cranberry, walnuts	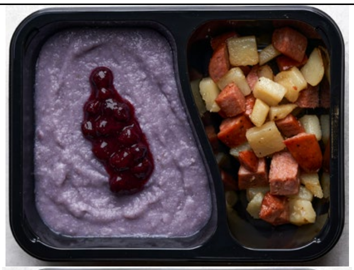 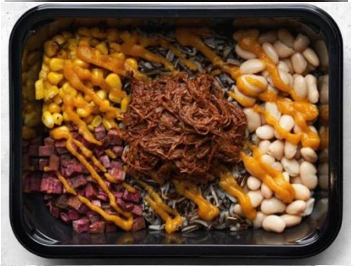 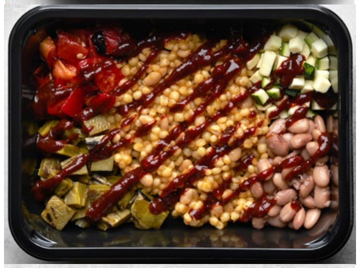 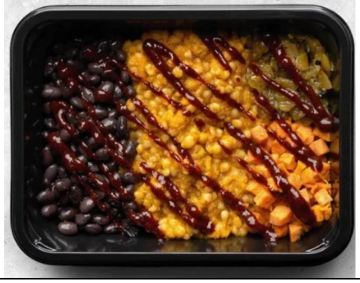
	Blue Corn Mush Plate	Blue corn meal, blueberries, bison sausage, potatoes, butternut squash	
	Blue Corn Pancake and Hash	Blue corn meal, maple syrup, bison sausage, potatoes, butternut squash	
	Cherry Almond Oatmeal	Cherries, almonds, oatmeal, maple syrup	
	Green Chili Veggie Scramble	Eggs, green chili, Navajo ground beef, corn	
	Southwest Navajo Omelet Plate	Eggs, apples, blueberries, New Mexico green chili sauce	
	Blueberry Coconut Oatmeal	Blueberry, Oatmeal, Coconut	
7 lunch/ dinners	Pumpkin Neeshjizhii Stew	Pumpkin, Neeshjizhii (dry steamed corn), blue corn meal, wild rice	
	Shredded Bison Wheatberry Bowl	Bison, wheat berries, butternut squash, New Mexico chili sauce	
	Three Sisters Chowder	Squash, beans, corn, potatoes, blue corn, wild rice	
	Navajo Beef with Pasta	Butternut squash, Tepary beans, Navajo beef, whole grain pasta	
	Mutton Rainbow Stew Dinner	Mutton, Steamed Corn, blue corn meal, wild rice	
	Garden Veggie Squash Enchilada Bowl	Beans, squash, New Mexico red chili, Corn Tortilla	
	Navajo Bounty Harvest Bowl	Naples, Green Chile, Butternut squash, Tepary beans, Wheatberries	

© 2026 Eberly LA et al. *JAMA Internal Medicine*.

Supplemental Table 2. Nutrient Fact Panels for a Sample of MUTTON-HF Meals

Sample of Breakfasts

Blue Corn Mush Breakfast

Nutrition Facts

1 serving

Serving size	1 Meal, 14 oz. (399g)
Amount per serving	
**Calories**	**270**
	**% Daily Value***
**Total Fat** 5g	**6%**
Saturated Fat 2.5g	**13%**
Trans Fat 0g	
**Cholesterol** 20mg	**7%**
**Sodium** 310mg	**13%**
**Total Carbohydrate** 47g	**17%**
Dietary Fiber 5g	**18%**
Total Sugars 14g	
Includes 8g Added Sugars	**16%**
**Protein** 11g	
Vitamin D 0mcg	0%
Calcium 130mg	10%
Iron 2mg	10%
Potassium 502mg	10%

*The % Daily Value tells you how much a nutrient in a serving of food contributes to a daily diet. 2,000 calories a day is used for general nutrition advice.

Blue Corn Pancake Breakfast

Nutrition Facts

1 serving

Serving size	1 Meal, 9.5 oz. (269g)
Amount per serving	
**Calories**	**310**
	**% Daily Value***
**Total Fat** 7g	**9%**
Saturated Fat 2.5g	**13%**
Trans Fat 0g	
**Cholesterol** 35mg	**12%**
**Sodium** 550mg	**24%**
**Total Carbohydrate** 53g	**19%**
Dietary Fiber 8g	**29%**
Total Sugars 27g	
Includes 15g Added Sugars	**30%**
**Protein** 10g	
Vitamin D 0mcg	0%
Calcium 150mg	10%
Iron 2mg	10%
Potassium 462mg	10%

*The % Daily Value tells you how much a nutrient in a serving of food contributes to a daily diet. 2,000 calories a day is used for general nutrition advice.

Chiiłchin Apple Harvest Oatmeal

Nutrition Facts

1 serving

Serving size	1 Meal, 10 oz. (283g)
Amount per serving	
**Calories**	**330**
	**% Daily Value***
**Total Fat** 9g	**12%**
Saturated Fat 2.5g	**13%**
Trans Fat 0g	
**Cholesterol** 5mg	**2%**
**Sodium** 90mg	**4%**
**Total Carbohydrate** 64g	**23%**
Dietary Fiber 7g	**25%**
Total Sugars 36g	
Includes 20g Added Sugars	**40%**
**Protein** 6g	
Vitamin D 0mcg	0%
Calcium 80mg	6%
Iron 1.6mg	8%
Potassium 330mg	8%

*The % Daily Value tells you how much a nutrient in a serving of food contributes to a daily diet. 2,000 calories a day is used for general nutrition advice.

Sample of Lunch/Dinners

Mutton Rainbow Stew

Nutrition Facts

1 serving

Serving size	1 Meal, 13.5 oz. (383g)
Amount per serving	
**Calories**	**310**
	**% Daily Value***
**Total Fat** 9g	**12%**
Saturated Fat 3.5g	**18%**
Trans Fat 0g	
**Cholesterol** 80mg	**27%**
**Sodium** 400mg	**17%**
**Total Carbohydrate** 34g	**12%**
Dietary Fiber 4g	**14%**
Total Sugars 5g	
Includes 1g Added Sugars	**2%**
**Protein** 25g	
Vitamin D 0.1mcg	0%
Calcium 110mg	8%
Iron 3mg	15%
Potassium 540mg	10%

*The % Daily Value tells you how much a nutrient in a serving of food contributes to a daily diet. 2,000 calories a day is used for general nutrition advice.

Three Sisters Chowder and Cornbread

Nutrition Facts

1 serving

Serving size	1 Meal, 13.5 oz. (383g)
Amount per serving	
**Calories**	**350**
	**% Daily Value***
**Total Fat** 8g	**10%**
Saturated Fat 4g	**20%**
Trans Fat 0g	
**Cholesterol** 30mg	**10%**
**Sodium** 370mg	**16%**
**Total Carbohydrate** 61g	**22%**
Dietary Fiber 9g	**32%**
Total Sugars 8g	
Includes 1g Added Sugars	**2%**
**Protein** 12g	
Vitamin D 0.1mcg	0%
Calcium 130mg	10%
Iron 2.7mg	15%
Potassium 740mg	15%

*The % Daily Value tells you how much a nutrient in a serving of food contributes to a daily diet. 2,000 calories a day is used for general nutrition advice.

Pumpkin Neeshjizhii Stew

Nutrition Facts

1 serving

Serving size	1 Meal, 13.5 oz. (383g)
Amount per serving	
**Calories**	**370**
	**% Daily Value***
**Total Fat** 11g	**14%**
Saturated Fat 2g	**10%**
Trans Fat 0g	
**Cholesterol** 20mg	**7%**
**Sodium** 410mg	**18%**
**Total Carbohydrate** 65g	**24%**
Dietary Fiber 7g	**25%**
Total Sugars 21g	
Includes 14g Added Sugars	**28%**
**Protein** 8g	
Vitamin D 0.1mcg	0%
Calcium 150mg	10%
Iron 2.9mg	15%
Potassium 540mg	10%

*The % Daily Value tells you how much a nutrient in a serving of food contributes to a daily diet. 2,000 calories a day is used for general nutrition advice.

© 2026 Eberly LA et al. *JAMA Internal Medicine*.

Supplemental Figure 2. Map of Patient Communities, Study Sites and Food Program Sites

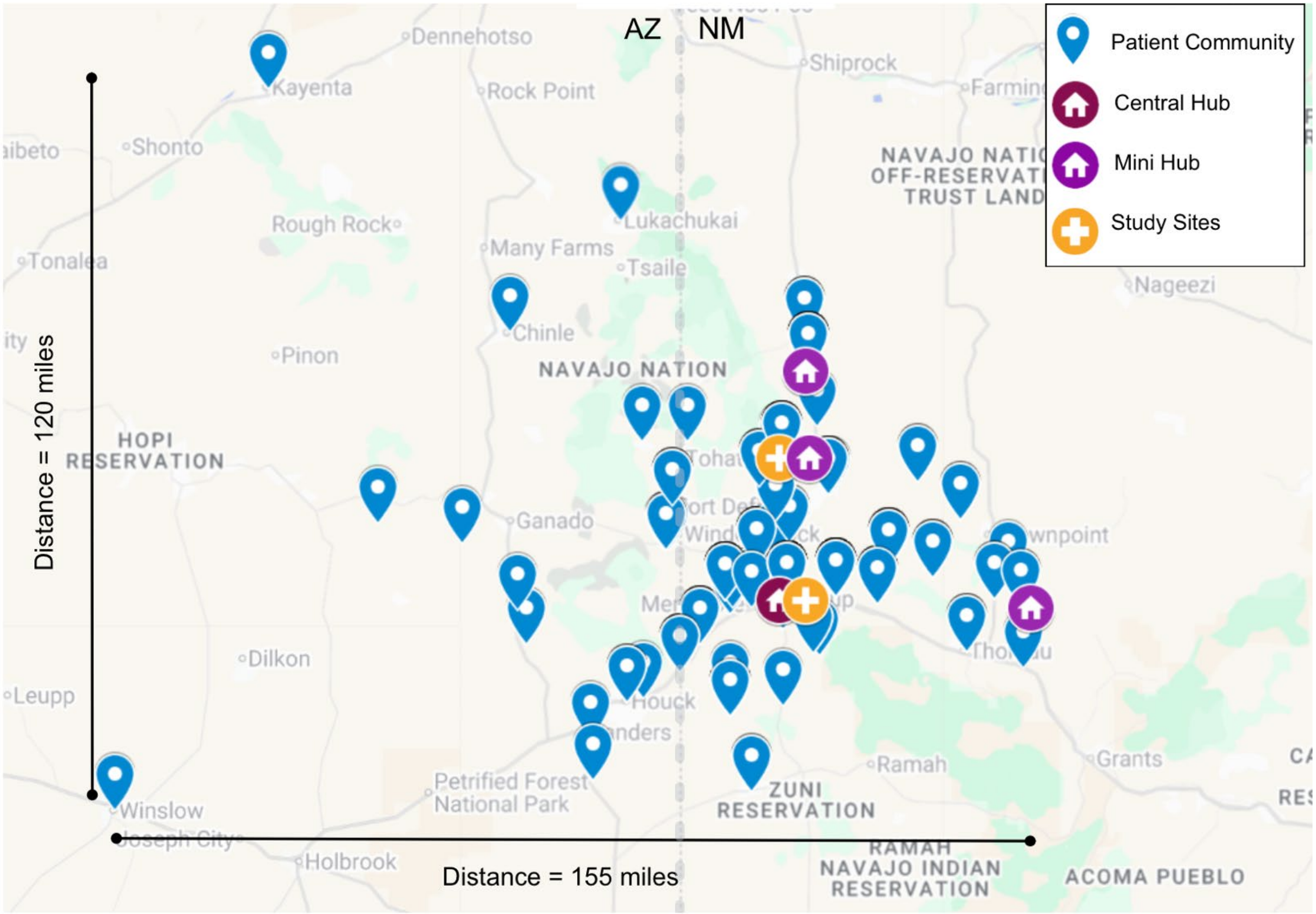

© 2026 Eberly LA et al. *JAMA Internal Medicine*.

Supplemental Table 3. Additional Baseline Characteristics by Treatment Arm

**Baseline Characteristic**	Intervention Arm (n=106)	Control Group (n=100)	Overall (n=206)
State of Residence, n (%)			
New Mexico	91 (85.8)	84 (84)	175 (85)
Arizona	15 (14.2)	16 (16)	31 (15)
Language Preferences, n (%)			
Speak English well	90 (92.8)	66 (81.5)	156 (87.6)
Speak Navajo well	73 (76)	59 (72.8)	132 (74.6)
Read English well	81 (83.5)	62 (76.5)	143 (80.3)
Read Navajo well	16 (16.7)	7 (8.6)	23 (13)
Cultural Connectedness Score, Traditions subscore, mean (SD)	6.5 (3.1)	5.9 (3.1)	6.2 (3.1)
Cultural Connectedness, Spirituality Subscore, mean (SD)	5.7 (1.9)	5.1 (2.1)	5.4 (2)
Cultural Connectedness Identity Subscore, mean (SD)	14.9 (3.6)	13.9 (4)	14.5 (3.8)
Eat Traditional Diné (Navajo) Meals, n (%)			

© 2026 Eberly LA et al. *JAMA Internal Medicine*.

1 time per last month	31 (32.3)	26 (32.5)	57 (32.4)
2-3 times last month	32 (33.3)	20 (25)	52 (29.5)
1 time per week	5 (5.2)	3 (3.8)	8 (4.5)
2 times per week	4 (4.2)	6 (7.5)	10 (5.7)
3-4 times per week	7 (7.3)	5 (6.3)	12 (6.8)
5-6 times per week	0 (0)	2 (2.5)	2 (1.1)
1 time per day	2 (2.1)	2 (2.5)	4 (2.3)
2 - 3 times per day	3 (3.1)	0 (0)	3 (1.7)
Never	10 (10.4)	13 (16.3)	23 (13.1)
Don't know or prefer not to answer	2 (2.1)	3 (3.8)	5 (2.8)
How often do you not have enough money for bills?			
Never	23 (23.7)	15 (18.8)	38 (21.5)
Rarely	20 (20.6)	21 (26.3)	41 (23.2)
Sometimes	36 (37.1)	29 (36.3)	65 (36.7)
Often	14 (14.4)	9 (11.3)	23 (13)
Always	4 (4.1)	6 (7.5)	10 (5.6)
Missing	9	20	29

© 2026 Eberly LA et al. *JAMA Internal Medicine*.

Self-Reported Health Status, n (%)			
Poor	6 (6.2)	17 (21)	23 (12.9)
Fair	37 (38.1)	29 (35.8)	66 (37.1)
Good	36 (37.1)	27 (33.3)	63 (35.4)
Very Good	11 (11.3)	6 (7.4)	17 (9.6)
Excellent	7 (7.2)	2 (2.5)	9 (5.1)
Minutes of Exercise Weekly, mean (SD)	84.3 (107.7)	123.9 (236.5)	102.5 (179.2)
**Medications**			
SGLT2i	73 (68.9)	60 (60)	133 (64.6)
Metformin	22 (20.8)	20 (20)	42 (20.4)
Beta-blocker	76 (71.7)	71 (71)	147 (71.4)
ACEi/ARB/ARNI	80 (75.5)	72 (72)	152 (73.8)
Loop diuretic	53 (50)	60 (60)	113 (54.9)
Thiazide diuretic	2 (1.9)	0 (0)	2 (1)
Insulin	34 (32.1)	29 (29)	63 (30.6)
MRA	54 (50.9)	47 (47)	101 (49)
Statin	74 (69.8)	70 (70)	144 (69.9)

© 2026 Eberly LA et al. *JAMA Internal Medicine*.

Ezetimibe	5 (4.7)	3 (3)	8 (3.9)
PCSK9 inhibitor	2 (1.9)	1 (1)	3 (1.5)
GLP1RA	35 (33)	29 (29)	64 (31.1)
Amlodipine	16 (15.1)	8 (8)	24 (11.7)
Sulfonylurea	1 (0.9)	2 (2)	3 (1.5)
Another Blood Pressure agent	11 (10.4)	7 (7)	18 (8.7)
Fish Oil	0 (0)	1 (1)	1 (0.5)
Other glycemic agent(s)	3 (2.8)	2 (2)	5 (2.4)
Vascepa	2 (1.9)	0 (0)	2 (1)
Aspirin	48 (45.3)	37 (37)	85 (41.3)

SGLT2-sodium-glucose cotransporter 2 inhibitor; ACEi-Angiotensin-converting enzyme inhibitor; ARB-angiotensin II receptor blocker; ARNI- Angiotensin Receptor-Neprilysin Inhibitors; MRA-mineralocorticoid antagonist; PSCK9-proprotein convertase subtilisin/kexin type 9.

© 2026 Eberly LA et al. *JAMA Internal Medicine*.

Supplemental Table 4. Baseline characteristics between those with complete vs. missing clinical biomarkers[a]

	No Missing Values (**N=167**)	Missing Clinical Biomarkers[a] (**N=42**)
**Demographics**		
Age, years, mean (SD)	65.4 [63.3, 67.6]	67.2 [62.4, 72.0]
Gender, n (%)		
Male	95 (57.9) [50.3, 65.2]	24 (57.1) [42.2, 70.9]
Female	69 (42.1) [34.8, 49.7]	18 (42.9) [29.1, 57.8]
Race, n (%)		
American Indian or Alaskan Native	162 (98.8) [95.7, 99.7]	41 (97.6) [87.7, 99.6]
American Indian or Alaskan Native & White	2 (1.2) [0.3, 4.3]	0
White	0	1 (2.4) [0.4, 12.3]
Tribal Affiliation, n (%)		
Navajo	156 (95.1) [90.7, 97.5]	39 (95.1) [83.9, 98.7]
Zuni	2 (1.2) [0.3, 4.3]	0
Navajo & Zuni	0	1 (2.4) [0.4, 12.6]
Other	6 (3.7) [1.7, 7.8]	1 (2.4) [0.4, 12.6]
Ethnicity, n (%)		
Hispanic	3 (1.8) [0.6, 5.2]	0
Cultural Connectiveness Score Total, mean (SD)	26.1 [24.8, 27.4]	26.2 [23.3, 29.2]
Indigenous Nourishment Scale, mean (SD)	3.8 [3.6, 4.0]	3.6 [3.0, 4.2]
**Diet Quality and Characteristics**		
Responsible for some or most of the cooking	58 (39.2) [31.7, 47.2]	11 (36.7) [21.9, 54.5]
Daily Servings of Fruits and Vegetables (Based on DSQ-10), in cups, mean (SD)	2.5 [2.4, 2.7]	2.4 [2.1, 2.8]
**Socioeconomic and Household Characteristics**		
USDA Food Security Status, n (%)		
High food security	58 (39.7) [32.2, 47.8]	8 (26.7) [14.2, 44.4]
Low food security	59 (40.4) [32.8, 48.5]	15 (50.0) [33.2, 66.8]

© 2026 Eberly LA et al. *JAMA Internal Medicine*.

Very Low security	29 (19.9) [14.2, 27.1]	7 (23.3) [11.8, 40.9]
Financial Stress Score, mean (SD)	7.2 [6.7, 7.6]	7.2 [6.5, 7.9]
Working Microwave	129 (78.7) [71.8, 84.2]	25 (59.5) [44.5, 73.0]
Working Stove	137 (83.5) [77.1, 88.4]	29 (69.0) [54.0, 80.9]
Working Freezer	130 (79.3) [72.4, 84.8]	26 (61.9) [46.8, 75.0]
Working Refrigerator	136 (82.9) [76.4, 87.9]	27 (64.3) [49.2, 77.0]
Reliable Electricity	134 (81.7) [75.1, 86.9]	29 (69.0) [54.0, 80.9]
Running Tap water	121 (73.8) [66.6, 79.9]	27 (64.3) [49.2, 77.0]
Water Insecure*	73 (44.5) [37.1, 52.2]	22 (52.4) [37.7, 66.6]
Enrollment in Food Assistance Programs**		
Supplemental Nutrition Assistance Program/Food Stamps	58 (35.4) [28.5, 42.9]	17 (40.5) [27.0, 55.5]
Commodities	7 (4.3) [2.1, 8.5]	2 (4.8) [1.3, 15.8]
WIC	6 (3.7) [1.7, 7.8]	1 (2.4) [0.4, 12.3]
Other	61 (37.2) [30.2, 44.8]	14 (33.3) [21.0, 48.4]
**Clinical Characteristics**		
Weight, pounds, mean (SD)	187.6 [179.2, 196.0]	193.6 [174.7, 212.5]
Body Mass Index, kg/m^{2}, mean (SD)	30.3 [29.1, 31.5]	32 [29.2, 34.9]
Systolic blood pressure, mmHg, mean (SD)	129.9 [126.9, 133.0]	133.1 [127.2, 139.1]
Diastolic blood pressure, mmHg, mean (SD)	76.1 [74.1, 78.1]	77.5 [72.4, 82.6]
Type of Heart Failure, n (%)		
Ischemic	47 (28.7) [22.3, 36.0]	8 (19.0) [10.0, 33.3]
Nonischemic	60 (36.6) [29.6, 44.2]	17 (40.5) [27.0, 55.5]
Mixed	6 (3.7) [1.7, 7.8]	0
Unknown	51 (31.1) [24.5, 38.5]	17 (40.5) [27.0, 55.5]
Left Ventricular Ejection Fraction, percent, mean (SD)	47.9 [45.7, 50.1]	48.1 [44.2, 52.0]
Comorbidities, n (%)		
Hypertension	118 (72.0) [64.6, 78.3]	31 (73.8) [58.9, 84.7]
Atrial Fibrillation	33 (20.1) [14.7, 26.9]	13 (31.0) [19.1, 46.0]

© 2026 Eberly LA et al. *JAMA Internal Medicine*.

Coronary Artery Disease	78 (47.6)<br>[40.1, 55.2]	14 (33.3)<br>[21.0, 48.4]
Chronic Kidney Disease (CKD)	63 (38.4)<br>[31.3, 46.0]	9 (21.4)<br>[11.7, 35.9]
CKD on Hemodialysis	9 (5.5)<br>[2.9, 10.1]	1 (2.4)<br>[0.4, 12.3]
Type II Diabetes	111 (67.7)<br>[60.2, 74.4]	26 (61.9)<br>[46.8, 75.0]
Hyperlipidemia	107 (65.2)<br>[57.7, 72.1]	28 (66.7)<br>[51.6, 79.0]
Obesity	73 (44.5)<br>[37.1, 52.2]	22 (52.4)<br>[37.7, 66.6]
Prediabetes	34 (20.7)<br>[15.2, 27.6]	9 (21.4)<br>[11.7, 35.9]
Obstructive Sleep Apnea	39 (23.8)<br>[17.9, 30.8]	10 (23.8)<br>[13.5, 38.5]
KCCQ summary score total, mean (SD)	59.4 [55.5, 63.2]	53.5 [44.7, 62.4]
KCCQ physical limitation score, mean (SD)	55.5 [50.8, 60.2]	55.3 [44.1, 66.5]
KCCQ symptom frequency score, mean (SD)	66.3 [61.7, 70.9]	63 [52.1, 73.9]
KCCQ quality of life score, mean (SD)	56.3 [51.6, 61.0]	45.8 [34.1, 57.6]
KCCQ social limitation score, mean (SD)	58.7 [53.3, 64.0]	49.6 [39.2, 60.0]

[a] Missing 2 or more clinical biomarkers (given some patients did not provide urine sample for urine tests and some missing 1 clinical biomarker due to issues including laboratory assay stock outs, laboratory error in drawing all labs)
[b] P-values determined with Fisher exact test or t-test
[c] Water Insecurity determined by validated water security indicators with having any of the following responses being consistent with water insecurity: answering "no" to "Do you have running (tap) water"; answering "disagree" to "My tap water at home is safe to drink" or "My tap water at home is safe to cook with"; or "I never drink tap water" when asked "When you drink tap water, what is the main source of the tap water?" (Slotnick MJ, Leung CW. Water Insecurity Indicators Are Associated with Lower Diet and Beverage Quality in a National Survey of Lower-Income United States Adults. J Nutr. 2023 Nov;153(11):3308-3316. doi: 10.1016/j.tjnut.2023.08.019. Epub 2023 Aug 23. PMID: 37619920)
[d] In prior 12 months

© 2026 Eberly LA et al. *JAMA Internal Medicine*.

Supplemental Table 5. Baseline characteristics between those with complete vs. missing survey data

	No Missing Survey Data (**N=193**)	Missing Survey Data (**N=13**)
**Demographics**		
Age, years, mean (SD)	65.5 [63.5, 67.5]	69.8 [59.7, 79.9]
Gender, n (%)		
Male	112 (58.0) [51.0, 64.8]	7 (53.8) [29.1, 76.8]
Female	81 (42.0) [35.2, 49.0]	6 (46.2) [23.2, 70.9]
Race, n (%)		
American Indian or Alaskan Native	190 (98.4) [95.5, 99.5]	13 (100.0) [77.2, 100.0]
American Indian or Alaskan Native & White	2 (1.0) [0.3, 3.7]	0
White	1 (0.5) [0.1, 2.9]	0
Tribal Affiliation, n (%)		
Navajo	183 (94.8) [90.7, 97.2]	12 (92.3) [66.7, 98.6]
Zuni	2 (1.0) [0.3, 3.7]	0
Navajo & Zuni	1 (0.5) [0.1, 2.9]	0
Other	6 (3.1) [1.4, 6.6]	1 (7.7) [1.4, 33.3]
Ethnicity, n (%)		
Hispanic	3 (1.6) [0.5, 4.5]	0
Cultural Connectiveness Score Total, mean (SD)	26.4 [25.2, 27.6]	21.7 [16.8, 26.6]
Indigenous Nourishment Scale, mean (SD)	3.8 [3.6, 4.1]	3 [1.9, 4.0]
**Diet Quality and Characteristics**		
Responsible for some or most of the cooking	68 (40.5) [33.3, 48.0]	1 (10.0) [1.8, 40.4]
Daily Servings of Fruits and Vegetables (Based on DSQ-10), in cups, mean (SD)	2.5 [2.4, 2.6]	2.7 [2.0, 3.3]
**Socioeconomic and Household Characteristics**		
USDA Food Security Status, n (%)		
High food security	60 (36.1) [29.2, 43.7]	6 (60.0) [31.3, 83.2]
Low food security	71 (42.8) [35.5, 50.4]	3 (30.0) [10.8, 60.3]
Very Low security	35 (21.1) [15.6, 27.9]	1 (10.0) [1.8, 40.4]
Financial Stress Score, mean (SD)	7.3 [6.9, 7.7]	5.3 [3.4, 7.2]
Working Microwave	145 (75.1) [68.6, 80.7]	9 (69.2) [42.4, 87.3]
Working Stove	156 (80.8) [74.7, 85.8]	10 (76.9) [49.7, 91.8]
Working Freezer	148 (76.7)	8 (61.5)

© 2026 Eberly LA et al. *JAMA Internal Medicine*.

	[70.2, 82.1]	[35.5, 82.3]
Working Refrigerator	153 (79.3) [73.0, 84.4]	10 (76.9) [49.7, 91.8]
Reliable Electricity	153 (79.3) [73.0, 84.4]	10 (76.9) [49.7, 91.8]
Running Tap water	139 (72.0) [65.3, 77.9]	9 (69.2) [42.4, 87.3]
Water Insecure*	88 (45.6) [38.7, 52.6]	7 (53.8) [29.1, 76.8]
Enrollment in Food Assistance Programs**		
Supplemental Nutrition Assistance Program/Food Stamps	74 (38.3) [31.8, 45.4]	1 (7.7) [1.4, 33.3]
Commodities	9 (4.7) [2.5, 8.6]	0
WIC	6 (3.1) [1.4, 6.6]	1 (7.7) [1.4, 33.3]
Other	71 (36.8) [30.3, 43.8]	4 (30.8) [12.7, 57.6]
**Clinical Characteristics**		
Weight, pounds, mean (SD)	189.2 [181.3, 197.2]	181.9 [152.4, 211.3]
Body Mass Index, kg/m^2, mean (SD)	30.6 [29.5, 31.7]	31.5 [25.2, 37.9]
Systolic blood pressure, mmHg, mean (SD)	130.3 [127.5, 133.1]	134.4 [124.7, 144.1]
Diastolic blood pressure, mmHg, mean (SD)	76.7 [74.7, 78.6]	71.5 [63.7, 79.3]
Type of Heart Failure, n (%)		
Ischemic	51 (26.4) [20.7, 33.1]	4 (30.8) [12.7, 57.6]
Nonischemic	72 (37.3) [30.8, 44.3]	5 (38.5) [17.7, 64.5]
Mixed	6 (3.1) [1.4, 6.6]	0
Unknown	64 (33.2) [26.9, 40.1]	4 (30.8) [12.7, 57.6]
Left Ventricular Ejection Fraction, percent, mean (SD)	47.9 [45.9, 49.8]	49.3 [39.5, 59.2]
Comorbidities, n (%)		
Hypertension	140 (72.5) [65.8, 78.3]	9 (69.2) [42.4, 87.3]
Atrial Fibrillation	43 (22.3) [17.0, 28.7]	3 (23.1) [8.2, 50.3]
Coronary Artery Disease	86 (44.6) [37.7, 51.6]	6 (46.2) [23.2, 70.9]
Chronic Kidney Disease (CKD)	64 (33.2) [26.9, 40.1]	8 (61.5) [35.5, 82.3]
CKD on Hemodialysis	10 (5.2) [2.8, 9.3]	0
Type II Diabetes	127 (65.8) [58.9, 72.1]	10 (76.9) [49.7, 91.8]

© 2026 Eberly LA et al. *JAMA Internal Medicine*.

Hyperlipidemia	127 (65.8)<br>[58.9, 72.1]	8 (61.5)<br>[35.5, 82.3]
Obesity	89 (46.1)<br>[39.2, 53.2]	6 (46.2)<br>[23.2, 70.9]
Prediabetes	39 (20.2)<br>[15.1, 26.4]	4 (30.8)<br>[12.7, 57.6]
Obstructive Sleep Apnea	44 (22.8)<br>[17.4, 29.2]	5 (38.5)<br>[17.7, 64.5]
KCCQ summary score total, mean (SD)	58.6 (24)	54.5 (21.4)
KCCQ physical limitation score, mean (SD)	55.7 (28.8)	51.9 (23.9)
KCCQ symptom frequency score, mean (SD)	65.4 (28.8)	70.8 (22.4)
KCCQ quality of life score, mean (SD)	54.8 (29.9)	50 (24.3)
KCCQ social limitation score, mean (SD)	57.9 (31.3)	40.6 (33.2)

[a] P-values determined with Fisher exact test or t-test
[b]Water Insecurity determined by validated water security indicators with having any of the following responses being consistent with water insecurity: answering “no” to “Do you have running (tap) water”; answering “disagree” to “My tap water at home is safe to drink” or “My tap water at home is safe to cook with”; or “I never drink tap water” when asked “When you drink tap water, what is the main source of the tap water?” (Slotnick MJ, Leung CW. Water Insecurity Indicators Are Associated with Lower Diet and Beverage Quality in a National Survey of Lower-Income United States Adults. J Nutr. 2023 Nov;153(11):3308-3316. doi: 10.1016/j.tjnut.2023.08.019. Epub 2023 Aug 23. PMID: 37619920)
[c]In prior 12 months

© 2026 Eberly LA et al. *JAMA Internal Medicine*.

Supplemental Table 6. Implementation Characteristics Among those in the Intervention Arm (n=106)

Implementation Outcome	Intervention Arm (n=106)
Meal boxes picked up, Mean (SD)	6.0 (2.7)
% of meal boxes picked up, Mean (SD)	75.6 (33.2)
Program recommendation	
Score, Mean (SD)	8.3 (2.3)
Net Promoter Score	39%
Percent of meal consumed	
None	3 (3.0)
About a quarter/some (25%)	10 (10.0)
About half (50%)	12 (12.0)
About three quarters/most (75%)	42 (42.0)
All or nearly all (90-100%)	33 (33.0)

Missing: 8 for program ranking and Net Promoter score, 9 for amount of meals consumed.

© 2026 Eberly LA et al. *JAMA Internal Medicine*.

Supplemental Table 7. Distributed household appliances by group assignment

Appliance	Intervention Group (n=106)	Control Group (n=100)
Microwave	4 (2.9%)	7 (6.9%)
Mini refrigerator/freezer (electric)	6 (4.8 %)	5 (5.0%)
Propane-powered refrigerator plus propane tank (no electricity)	6 (5.7%)	2 (2.0%)
Blender*	1 (0.95%)	0 (0.0%)
**Total**	17 (16.0%)	14 (14.0%)

* To help soften meals to eat given denture issues.

© 2026 Eberly LA et al. *JAMA Internal Medicine*.

Supplemental Table 8. Adverse Events by Assigned Treatment Arm

Adverse Event	Intervention Arm (N=106)	Control Group (N=100)	Relative Risk	P-value
Another event other than hospitalization or emergency room visit requiring clinical care	10 (9.5)	6 (6)	1.48 (0.67, 3.27)	0.33
Heart Failure/Volume overload	9 (8.6)	5 (5)	1.77 (0.74, 4.25)	0.20
Food-borne illness	0	0		
Acute kidney injury	0	0		
Hyperkalemia	0	0		
Hypoglycemia	0	0		
Hyponatremia	0	0		
Hypokalemia	0	0		
Hyperglycemia	0	0		
Hypertension (SBP >180)	1 (1)	0 (0)		
Hypotension (SBP < 90)	1 (1)	0 (0)		
Death	0	0		
Other*	11 (10.5)	7 (7)		
Other event NOT requiring clinical care	3 (2.9)	3 (3)	0.52 (0.20, 1.35)	0.18
Heart Failure/Volume overload	6 (5.7)	12 (12)	0.48 (0.19, 1.22)	0.12
Food-borne illness	0	0		
Acute kidney injury	1 (1)	2 (2)		
Hyperkalemia	1 (1)	2 (2)		
Hypoglycemia	0	0		
Hyponatremia	0 (0)	1 (1)		
Hypokalemia	0	0		

© 2026 Eberly LA et al. *JAMA Internal Medicine*.

Hyperglycemia	1 (1)	0 (0)		
Hypertension (SBP >180)	0 (0)	2 (2)		
Hypotension (SBP < 90)	0 (0)	2 (2)		
Death	0 (0)	1 (1)		
Other**	3 (2.9)	2 (2)		

* Other events requiring clinical care include the following in the intervention group: atopic dermatitis acute flare, balanitis, COVID infection, wheezing/asthma exacerbation, foot callus, headache, inguinal hernia, lower extremity cellulitis (2), low back pain, motor vehicle accident, tinnitus, urinary tract infection; and the following in control group: foot blister, knee pain, otitis media, sore throat, urinary tract infection, viral URI, vitreous hemorrhage.

**Other events not requiring clinical care include the following in the intervention arm: elbow injury, hyperphosphatemia, and sinusitis; and the following in the control group: leg pain and vaginal candidiasis.

© 2026 Eberly LA et al. *JAMA Internal Medicine*.

Supplemental Table 9. Total Events and Outcome Counts by Assigned Treatment Arm

	Intervention Arm (N=106)	Control Group (N=100)	Incidence Rate Ratio (95% CI)	P-value
**Hospitalization or ER visit, total count**	82	110	0.71 (0.43, 1.16)	0.15
**Hospitalization (all-cause), total count**	20	41	0.46 (0.24, 0.91)	0.02
**ER Visit (all cause), total count**	62	69	0.86 (0.49, 1.49)	0.57
**Hospitalization or ER visit for HF**	5	15	0.32 (0.10, 1.00)	0.05
**HF Hospitalization**	4	14	0.27 (0.09, 0.81)	0.02
**ER visit for HF**	1	1	0.95 (0.06, 15.02)	0.97

ER-emergency room; HF-heart failure

© 2026 Eberly LA et al. *JAMA Internal Medicine*.

Supplemental Table 10. Exploratory Outcomes

Outcome	Arm	Baseline	Post Baseline	Change	Percent change	P-Value
**Minutes of Exercise Weekly**						
	Food arm	84.27 (107.73)	147.78 (197.04)	66.44 (202.06)	594.35 (2574.17)	0.03
	Control	123.91 (236.45)	82.37 (99.98)	-63.71 (252.84)	387.82 (1302.74)	0.00
	Food vs. control			130.15 (226.77)	206.53 (2089.40)	0.003
**Self-Reported Health Status***						
	Food arm					
	Excellent	7 (7.2)	10 (9.9)			0.04
	Very Good	11 (11.3)	13 (12.9)			
	Good	36 (37.1)	49 (48.5)			
	Fair	37 (38.1)	21 (20.8)			
	Poor	6 (6.2)	8 (7.9)			
	Control Group					
	Excellent	2 (2.5)	1 (1.1)			0.18
	Very Good	6 (7.4)	10 (11.4)			
	Good	27 (33.3)	30 (34.1)			
	Fair	29 (35.8)	38 (43.2)			
	Poor	17 (21)	9 (10.2)			
	Food vs. control					0.29

© 2026 Eberly LA et al. *JAMA Internal Medicine*.
