# Supplementary material for: An Indigenous Food Is Medicine Intervention: The MUTTON-HF Randomized Clinical Trial
Source: JAMA Intern Med. 2026 Jul 27:e262879. Online ahead of print. doi: 10.1001/jamainternmed.2026.2879 (PMC13409115; doi:10.1001/jamainternmed.2026.2879)
Supplement: Supplement 3. — Data sharing statement [file jamainternmed-e262879-s003.pdf]

Data Sharing Statement

Eberly. An Indigenous Food Is Medicine Intervention. *JAMA Intern Med*. Published July 27, 2026. doi:10.1001/jamainternmed.2026.2879

Data
Additional Information: Clinicaltrials.gov: https://clinicaltrials.gov/study/NCT06549699 NCT06549699
Data available: No

Additional Information
Explanation for why data not available: Per tribal regulations, data cannot be shared, but can be requested by the Navajo Nation Human Research Review Board.
